# Assessing the reduction of viral infectivity in HPV16/18-positive women after one, two, and three doses of Gardasil-9 (RIFT): Study protocol

**DOI:** 10.1371/journal.pone.0304080

**Published:** 2024-05-20

**Authors:** Victoria López-Codony, Álvaro de Andrés-Pablo, Angelica Ferrando-Díez, Maria Eulàlia Fernández-Montolí, Marta López-Querol, Sara Tous, Carlos Ortega-Expósito, Juan Carlos Torrejón-Becerra, Yolanda Pérez, Anna Ferrer-Artola, Josep Maria Sole-Sedeno, Clara Grau, Blas Rupérez, Maria Saumoy, Mónica Sánchez, Paula Peremiquel-Trillas, Laia Bruni, Laia Alemany, Francesc Xavier Bosch, Miquel Angel Pavón

**Affiliations:** 1 Catalan Institute of Oncology, Bellvitge Biomedical Research Institute (IDIBELL), Cancer Epidemiology Research Programme, L’Hospitalet de Llobregat, Barcelona, Spain; 2 Programa de Doctorat en Biomedicina, Universitat de Barcelona (UB), Barcelona, Spain; 3 Medical Oncology Department, Catalan Institute of Oncology, Germans Trias i Pujol University Hospital (HGTiP), Badalona, Barcelona, Spain; 4 Department of Gynaecology, Bellvitge University Hospital (HUB), L’Hospitalet de Llobregat, Barcelona, Spain; 5 Biomedical Research Networking Center for Epidemiology and Public Health (CIBERESP), Madrid, Spain; 6 Bellvitge Biomedical Research Institute (IDIBELL), Pharmacy Unit, Bellvitge University Hospital (HUB), L’Hospitalet de Llobregat, Barcelona, Spain; 7 Department of Obstetrics and Gynaecology, Hospital del Mar–Mar Health Park, Barcelona, Spain; 8 Sexual and Reproductive Health Care Center–ASSIR, Delta del Llobregat, Barcelona, Spain; 9 HIV and STD Unit, Bellvitge University Hospital (HUB), Bellvitge Biomedical Research Institute (IDIBELL), L’Hospitalet de Llobregat, Barcelona, Spain; 10 Faculty of Health Sciences, Open University of Catalonia (UOC), Barcelona, Spain; University of Michigan, UNITED STATES

## Abstract

Human Papillomavirus (HPV) prophylactic vaccination has proven effective in preventing new infections, but it does not treat existing HPV infections or associated diseases. Hence, there is still an important reservoir of HPV in adults, as vaccination programs are mainly focused on young women. The primary objective of this non-randomized, open-label trial is to evaluate if a 3-dose regimen of Gardasil-9 in HPV16/18-positive women could reduce the infective capacity of their body fluids. We aim to assess if vaccine-induced antibodies could neutralize virions present in the mucosa, thus preventing the release of infective particles and HPV transmission to sexual partners. As our main endpoint, the E1^E4-HaCaT model will be used to assess the infectivity rate of cervical, anal and oral samples, obtained from women before and after vaccination. HPV DNA positivity, virion production, seroconversion, and the presence of antibodies in the exudates, will be evaluated to attribute infectivity reduction to vaccination. Our study will recruit two different cohorts (RIFT-HPV1 and RIFT-HPV2) of non-vaccinated adult women. RIFT-HPV1 will include subjects with an HPV16/18 positive cervical test and no apparent cervical lesions or cervical lesions eligible for conservative treatment. RIFT-HPV2 will include subjects with an HPV16/18 positive anal test and no apparent anal lesions or anal lesions eligible for conservative treatment, as well as women with an HPV16/18 positive cervical test and HPV-associated vulvar lesions. Subjects complying with inclusion criteria for both cohorts will be recruited to the main cohort, RIFT-HPV1. Three doses of Gardasil-9 will be administered intramuscularly at visit 1 (0 months), visit 2 (2 months) and visit 3 (6 months). Even though prophylactic HPV vaccines would not eliminate a pre-existing infection, our results will determine if HPV vaccination could be considered as a new complementary strategy to prevent HPV-associated diseases by reducing viral spread.

**Trial registration**: https://clinicaltrials.gov/ct2/show/NCT05334706.

## Introduction

Worldwide, more than 610,000 cancer cases are annually attributed to Human Papillomavirus (HPV), including most cervical cancers and several anogenital (vulvar, vaginal, penile, and anal) and oropharyngeal cancers [1,2]. Out of all the HPV genotypes, HPV16 is the most carcinogenic type and both HPV16 and HPV18 are responsible for more than 70% of cervical cancers [1,2].

The 9vHPV vaccine (Gardasil-9) consists of highly purified virus-like particles (VLPs) of the recombinant major capsid (L1) protein of HPV Types 6, 11, 16, 18, 31, 33, 45, 52, and 58 [3]. The mechanism of protection has not been fully elucidated but it is largely accepted that vaccine-induced neutralizing antibodies (nAbs) play a crucial role in preventing the binding of virions to the epithelial cell surface, inhibiting the initiation of a new viral infection [4,5]. Moreover, it has been demonstrated that nAbs-labelled virions are targeted for proteasomal degradation by tripartite motif-containing protein 21 (*TRIM21*) [6]. Therefore, when HPV is transmitted to a vaccinated person, vaccine-induced nAbs can recognise and neutralize HPV virions, thus preventing epithelial infection. However, if the infection was acquired before vaccination, nAbs will not be able to eliminate infected cells.

Given that L1-VLP vaccines are prophylactic and not therapeutic, HPV vaccination programs are mainly aimed at adolescents before their first sexual encounter [7]. By excluding adults from vaccination strategies, individuals with a productive infection become an important reservoir of HPV, facilitating transmission to new individuals and HPV perpetuation in the population [8]. It has been demonstrated that anti-L1 nAbs are present in cervical and oral fluids, as well as in first-void urine samples, after seroconversion [9–20]. This suggests that in seroconverted individuals with a productive infection anti-L1 nAbs could interact with HPV virions, potentially neutralizing their infectivity [21]. Considering vaccine-induced antibody responses are significantly higher than natural serological responses, vaccination could enhance virion neutralization, limiting HPV transmission [21,22]. Based on this, recent vaccine trials have set out to evaluate if late vaccination could reduce HPV transmission [23–26]. An evaluation of recently formed heterosexual couples from the HITCH prospective study showed that HPV transmission to their respective partners was significantly reduced in vaccinated women ranging from 18 to 24 years old [23,24]. Moreover, a lower viral load was observed in vaccinated women with HPV6/11/16/18 infections, when compared to unvaccinated women [23]. However, the preliminary results of the following interventional clinical trial, TRAP-HPV, showed inconclusive evidence regarding if HPV vaccination could reduce transmission and in turn protect sex partners from new vaccine-preventable infections [25,26].

Given the complexity and potential biases that come with clinical trials involving couples, our study takes on a new perspective that focuses on infectivity, instead of transmission to sexual partners. We aim to assess if transudated nAbs in the epithelial mucosa, as a result of vaccination, could neutralize the virions released from infected cells, thus preventing new infections and HPV transmission. Here, we propose a method to measure HPV infectivity based on the in vitro infection of human keratinocytes cells (HaCaT) and the subsequent expression of the HPV E1^E4 spliced transcript. E1^E4 mRNA is a surrogate marker of viral machinery activation, allowing us to evaluate the number of keratinocytes that have been infected [27–32]. Using this E1^E4-HaCaT model, our study sets out to evaluate if a 3-dose regimen of Gardasil-9 could reduce the infective capacity of body fluids from HPV16/18-positive women.

## Materials and methods

### Study design and setting

This non-randomized, open-label trial will assess if vaccination with a 3-dose regimen of Gardasil-9 could neutralize HPV virions and reduce the infectivity of body fluids (cervical, anal and oral) from HPV16/18-positive women. The primary objective will be to evaluate infectivity reduction in body fluids after 3-doses of Gardasil-9. The main endpoint will be to evaluate the in vitro infectivity rate, together with complementary endpoints such as HPV DNA detection and genotyping, HPV16/18 virion detection and anti-HPV L1 detection in body fluids. The secondary objective will be to analyse anti-HPV L1 antibody titration in serum before and after vaccination. The tertiary objective will be to evaluate the infectivity reduction after 1 or 2-doses of Gardasil-9, using the same endpoints as in the primary objective. The study is lead by the Cancer Epidemiology Research Program (PREC) at the Catalan Institute of Oncology (ICO) and subject recruitment is conducted at the Gynaecology Unit of Bellvitge University Hospital (HUB), both in L’Hospitalet del Llobregat, Barcelona. Four satellite sites–Obstetrics and Gynaecology Department of Hospital del Mar at Mar Health Park in Barcelona; Sexual and Reproductive Health Care Center (ASSIR) in Delta del Llobregat, Barcelona; HIV and STD Unit at HUB; and Cervical Cancer Screening Technical Office at ICO–will identify and refer potential candidates. More details can be found in the original protocol version (S1 and S2 Files).

### Study population and recruitment criteria

The trial population will be composed of non-vaccinated adult women (18 years or older) positive for HPV16 and/or HPV18. A minimum of 39 and 30 subjects will be enrolled in two different study cohorts, RIFT-HPV1 and RIFT-HPV2, respectively. The RIFT-HPV1 cohort, which aims to measure cervical sample infectivity, will include adult women with an HPV16 and/or HPV18 positive cervical test and no apparent cervical lesions or cervical intraepithelial neoplasia (CIN) 1/2 lesions, eligible for conservative treatment. Alternatively, the RIFT-HPV2 cohort, which aims to measure anal sample infectivity, will include adult women with an HPV16 and/or HPV18 positive anal test and no apparent anal lesions or anal lesions eligible for conservative treatment. Additionally, the RIFT-HPV2 cohort will also include adult women with an HPV16 and/or HPV18 positive cervical test and HPV-associated vulvar lesions that would have been excluded from the RIFT-HPV1 cohort due to the need for surgical treatment. Although this cohort can overlap with the RIFT-HPV1 cohort, it will allow us to enrich the number of subjects with anal infection, reaching the sample size needed to evaluate vaccination impact on anal mucosa infectivity. In the event of identifying subjects that comply with inclusion criteria for both cohorts, they will be recruited to the main cohort, RIFT-HPV1. The impact of vaccination on cervical sample infectivity will be measured in patients included in RIFT-HPV1 cohort where cervical mucosa will not be altered by any surgical procedures. Alternatively, the vaccination impact on anal sample infectivity will be measured combining data from both cohorts, including all those women with an HPV16 and/or HPV18 positive anal test and no anal lesions or anal lesions that have not undergone any surgical treatment that could alter the anal mucosa. Recruitment started on September 13, 2022 and the overall study will end when the last subject completes the last study-related telephone-call or visit, withdraws from the study, or is lost to follow-up.

#### Inclusion criteria

To be eligible for the study, subjects must be adult women participating at cervical cancer screening or attending a routine gynaecological outpatient visit. They must be positive for HPV16, 18 or double-positive for 16 and 18; have been recently diagnosed for their HPV-positivity (within the last 10 months); and meet one of the previously stated criteria from RIFT-HPV1 or RIFT-HPV2 cohorts. Coinfection with other non HPV16/18 types, as well as history of cervical surgery, condyloma acuminate, HIV, transplant immunosuppression or an autoimmune disease, will not be considered exclusion criteria.

#### Exclusion criteria

Reasons for exclusion from the study include any cervical (RIFT-HPV1) or anal (RIFT-HPV2) lesion that requires clinical intervention within 7 months, history of severe allergic reaction or known allergy to any vaccine component, thrombocytopenia or any coagulation disorder, splenectomy, anogenital cancer or HPV-related head and neck cancer, pregnancy, previous receipt of any HPV vaccine, receipt of any immune globulin product or blood-derived product 3 months prior to the Day 1, receipt of inactivated or recombinant vaccines within 14 days prior to Day 1, or live vaccines within 21 days prior to Day 1.

#### Discontinuation criteria

Subjects may discontinue vaccination at any time for any reason or be discontinued at the discretion of the Investigator should any untoward effect occur. In addition, a subject may be discontinued by the Investigator or the Sponsor if study vaccination is inappropriate, the study plan is violated, or for administrative and/or other safety reasons. Discontinued subjects may resume the study vaccination regime if considered clinically safe and appropriate by the Investigator. Given that certain data beyond study treatment discontinuation may be important to the study, subjects will continue to be monitored after discontinuing treatment.

### Study procedures

**Fig 1:**
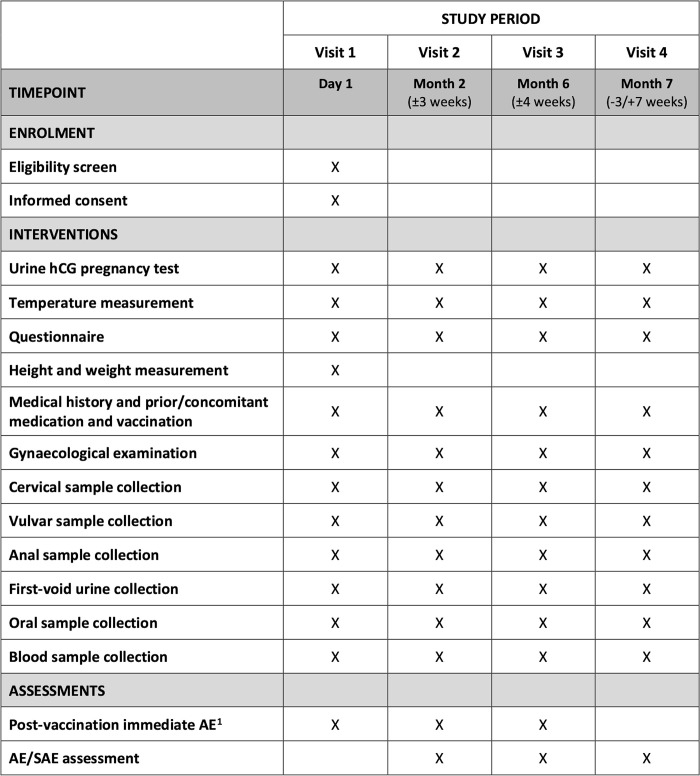
SPIRIT schedule of enrolment, interventions, and assessments. Abbreviations: hCG = human chorionic gonadotropin; AE = adverse events; SAE = severe adverse events. ^**1**^ All subjects will be observed by the Investigator or qualified designee for at least 30 minutes after each study vaccination for any untoward effects, including allergic reactions.

All enrolled subjects will complete the specified procedures in four visits (0, 2, 6, 7 months) (Fig 1). A pregnancy test will be performed on a urine sample in all four visits, before receiving any vaccine or collecting any other sample.

#### Study questionnaire

Study subjects will be required to answer a short questionnaire in all four visits, containing 10 questions regarding previous and current health status, HPV infections and vaccination, contraception use and sexual activity (S3 File).

#### Vaccine administration

Intramuscular Gardasil-9 vaccine will be administered as a 3-dose regimen in visits 1 to 3 by a qualified health professional following manufacturer’s protocol. Visits 2 and 3 will have an administration window period of ±3 and ±4 weeks, respectively.

#### Sample collection

A set of different samples, including cervical, anal, vulvar, oral, urine and blood samples, will be collected in visits 1 to 3, before vaccine administration, and in visit 4. Cervical samples will be collected using a Cervex-Brush® (Rovers Medical Devices B.V., North Brabant, Netherlands), which will be immediately immersed in a sterile collection tube containing 10 ml of phosphate-buffered saline (PBS). Anal samples will be collected using an endocervical brush (Bexen Medical, Gipuzkoa, Spain) and immediately immersed in 5 ml of PBS. Vulvar samples will be collected using a viscose collection swab (Deltalab S.L., Barcelona, Spain) which will be immersed in 5 ml of PBS upon arrival. Oral samples will be collected by a 15-second rinse and 15-second gargle with 10 ml of PBS. First-void urine samples will be collected using Colli-Pee® devices (Novosanis NV, Antwerp, Belgium), obtaining around 20ml of urine that will be split into two 15 ml tubes, one containing 4ml of UAS preservative medium (Novosanis NV, Antwerp, Belgium) and the other one empty. Blood samples will be extracted in a red-top vacutainer tube by standard venipuncture procedures and mixed by gently inverting the tube 5 times. Blood tubes will be left for one hour at room temperature for coagulation, after which they will be centrifuged at 1500 g for 10 minutes to separate serum from blood cells. All samples will be aliquoted in 1,5 ml tubes and stored at -80°C, except for a couple of aliquots that will be stored at 4–10°C for immediate usage (infectivity assay and genotyping test).

### Laboratory procedures

#### In vitro evaluation of viral infectivity

**Fig 2:**
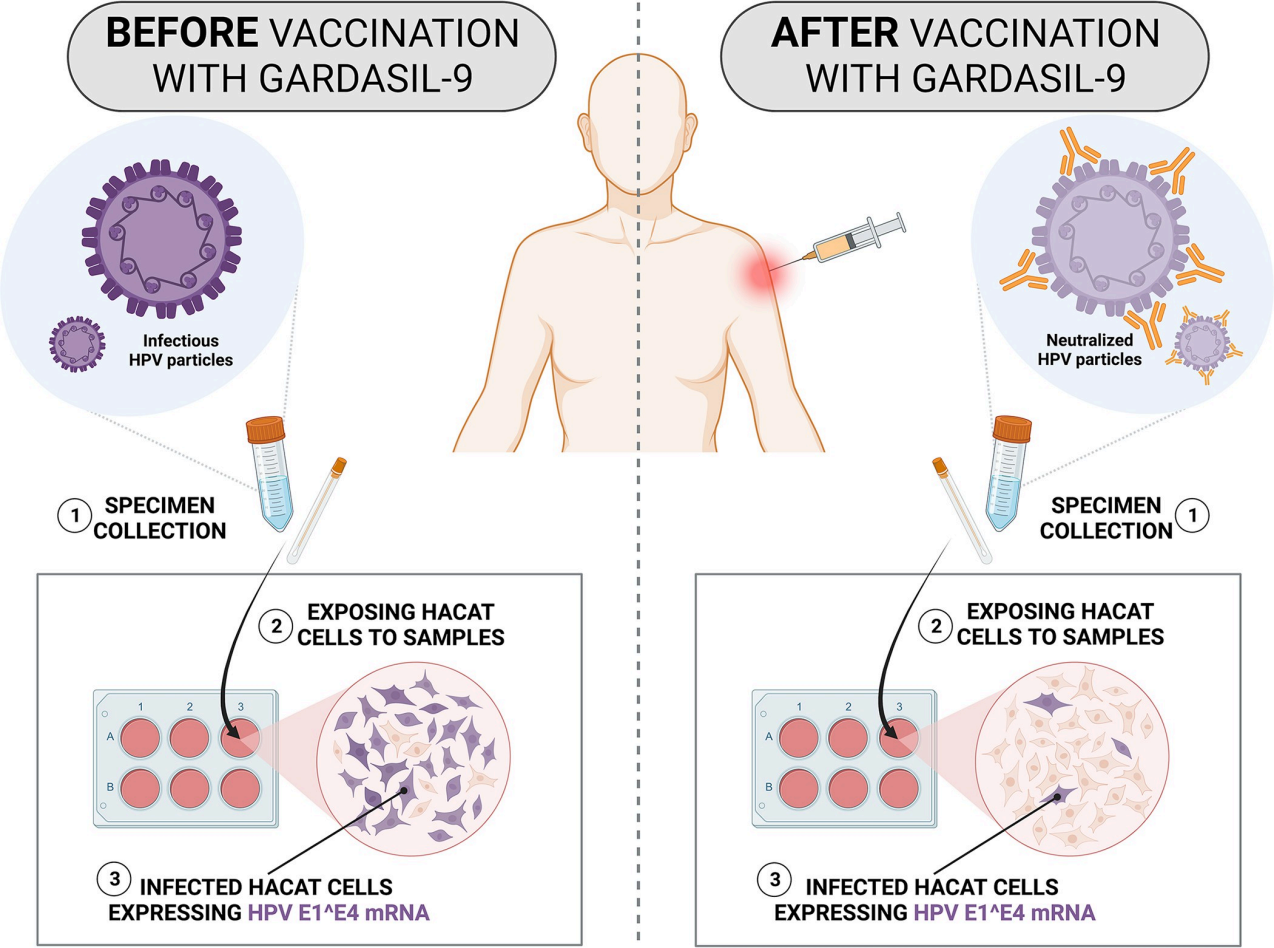
Infectivity assay performed on HaCaT cells before and after vaccination with Gardasil-9. Created with BioRender.com.

The E1^E4-HaCaT model is a functional assay that will allow us to indirectly quantify the presence of HPV virions in a sample, through the quantification of HPV E1^E4 mRNA expression in HaCaT cells after incubation with the collected specimens (cervical, anal and oral) (Fig 2). Quantification of HPV E1^E4 mRNA levels will be performed using two different gene expression assays: Quantitative reverse transcription PCR (RT-qPCR) and RNAscope *in situ* hybridization (RNAscope ISH). For the RT-qPCR assay, total RNA will be isolated from infected HaCaT cells using the PureLink^TM^ RNA Mini Kit (Thermo Fisher Scientific, Massachusetts, USA) and reverse transcribed to cDNA using the High-Capacity cDNA Reverse Transcription Kit (Thermo Fisher Scientific, Massachusetts, USA). E1^E4 mRNA will then be quantified on a CFX96 instrument (Bio-Rad Laboratories, Inc., California, USA) using TaqMan assays targeting HPV 16/18 E1^E4 (Thermo Fisher Scientific, Massachusetts, USA). For the ISH assay, infected HaCaT cells will be fixed and the RNAscope multiplex fluorescent assay will be performed using HPV 16/18 E1^E4 target probes (Advanced Cell Diagnostics, California, USA). Slides will be visualized under an inverted fluorescent microscope at 20-40X magnification.

#### HPV DNA detection and genotyping

In order to identify subjects undergoing natural clearance or new infections, HPV DNA detection and genotyping will be performed using the Anyplex II HPV28 assay (Seegene, Inc., Seoul, South Korea), identifying up to 28 HPV types (6, 11, 16, 18, 26, 31, 33, 35, 39, 40, 42, 43, 44, 45, 51, 52, 53, 54, 56, 58, 59, 61, 66, 68, 69, 70, 73 and 82), including the vaccine-preventable ones. DNA from cervical, anal and oral samples will be extracted using the automated Maxwell® RSC Tissue DNA Kit (Promega Corporation, Wisconsin, USA) according to manufacturer instructions.

#### HPV 16/18 virion detection

ELISA will be performed as primary method to detect and quantify HPV virions in cervical, anal and oral samples, in order to identify subjects with non-productive infections and distinguish them from subjects with productive infections but reduced infectivity due to vaccination. A sandwich ELISA assay will be used to detect the major capsid protein L1 of HPV 16/18 virions present in exudates of productive infections. Additionally, productive infections may be detected through the identification of virions by electron microscopy (EM), using the bioimaging platform services at Bellvitge Biomedical Research Institute (IDIBELL) [33,34].

#### Anti-HPV L1 Ab detection in cervical/anal/oral samples

ELISA will be performed to quantify anti-HPV L1 Ab against HPV 16 and/or 18 in cervical, anal, and oral samples from subjects, using microtiter plates pre-coated with our control HPV 16/18 PsV. This will enable us to associate the reduction in viral infectivity with the presence of neutralizing antibodies in these fluids.

#### Anti-HPV L1 Ab detection in serum

Serum samples will be shipped in dry ice to the accredited laboratory by Merck Sharp & Dohme (MSD) for anti-HPV L1 antibody titration (cLIA). The 9-valent HPV cLIA will be used to evaluate specific antibodies against HPV 6, 11, 16, 18, 31, 33, 45, 52, and 58 in serum samples from subjects after each Gardasil-9 dose [35]. This is a standardized technique commonly used in vaccine trials, which will also allow us to identify subjects with natural serological responses before vaccination.

### Study data management

All subjects signing the Informed Consent Form will be given a unique Study Identification Number (SIN), for purposes of identification in all procedures thorough the study, and anonymization for data analysis. Study database will be kept at all times in the study site’s designated digital store/internal server, which will be secured from unauthorized entry. Access will be exclusively permitted to study sponsor and statistician using password encryption. Upon completion of the study, data underlying the findings will be made publicly available in the Spanish Clinical Studies Registry (REec), as well as the ClinicalTrials.gov database, where it is registered.

### Safety considerations

Study subjects will be followed throughout the duration of the study for possible injection-site adverse events (AEs), systemic AEs, serious adverse event (SAEs) and deaths regardless of causality. All AEs and SAEs will be recorded by the study Investigator at each study visit. The Sponsor/representative will prepare and timely submit annual safety reports to the Spanish Agency of Medicines and Medical Devices (AEMPS) and the Clinical Research Ethics Committee of Bellvitge University Hospital (CRE-HUB), for the whole study’s duration.

### Statistical analysis

The primary endpoint will be the infectivity rate parameter, which will be measured by quantification of HPV-infected keratinocytes (i.e., expressing E1^E4 mRNA). The quantity of HPV-infected keratinocytes will be directly proportional to the quantity of free HPV virions present in samples (i.e., not neutralized by HPV antibodies). The expression levels of a positive control sample and a housekeeping human gene will be used to normalize the E1^E4 mRNA expression measurements. Differences in the infectivity rate of samples before and after the subject receives the first, second and third dose of Gardasil-9 will be compared using the non-parametric Wilcoxon signed rank test. Significant difference in the infectivity rate will be accepted with a p-value <0.05. ELISA virion detection, ELISA antibody detection and cLIA parameters will be included in the analysis to distinguish the effect of vaccination on the infectivity rate from other characteristics associated with the natural course of the HPV infection. Therefore, cases that after vaccination show a negative HPV16/18 DNA test, absence of HPV16/18 virions, absence of exudated anti-HPVL1 nAbs and/or absence of seroconversion, will not be included in the analysis population, as well as cases that in visit 1 show a negative HPV16/18 DNA test and/or absence of HPV16/18 virions.

#### Sample size and power

The sample size for the RIFT-HPV1 cohort was determined by using as reference the primary variable, which is the reduction in infectivity rates of cervical samples after three doses of Gardasil-9. With a significance level (alpha) of 0.05 and a statistical power (beta) of less than 0.2 in a two-tailed test, it was calculated that a minimum of 39 women receiving the three-dose vaccination regimen is required to detect a difference equal to or greater than a 25% reduction in the infectivity rate of cervical samples. For the RIFT-HPV2 cohort, the sample size was determined by using as reference the reduction in infectivity rates of anal samples after three doses of Gardasil-9. Using the same significance level and statistical power, it was estimated that a minimum of 28 women with an HPV-positive anal sample are needed for the three-dose vaccination schedule to detect a difference equal to or greater than a 35% reduction in the infectivity rate of anal samples. Recognizing that 28 of the women with HPV cervical infections may also have anal infections, 10 out of the 28 individuals included in the analysis will be drawn from the RIFT-HPV1 cohort. Considering that 60% of women showing multiple anogenital infection or with an anal previous positive test will show HPV positivity at study recruitment, we estimated that we need to recruit 30 new subjects in the RIFT-HPV2 cohort to add 18 new individuals in the analysis.

### Ethical considerations

The study protocol version 3.8 (November 11th, 2021), EudraCT number 2021-005229-26, received local Institutional Review Board (IRB) approval by CRE-HUB (AC032/21) on March 4^th^, 2022, as well as AEMPS (MUH/CLIN/EC) approval on March 9^th^, 2022. The current protocol version 4.1 (June 15th, 2023), including the latest amendments, received local IRB approval by CRE-HUB on October 11^th^, 2023, as well as AEMPS approval on October 17^th^, 2023. The study is being conducted in accordance with the principles of the Declaration of Helsinki, ICH Guidelines for Good Clinical Practice and local legislation (RD 1090/2015), and in full conformity with relevant regulations [36,37]. Written consent will be acquired from each participant upon execution of the Informed Consent Form, following which a distinct SIN will be assigned to ensure anonymity. The list of study subjects’ IDs and corresponding SINs will be kept by the study site under strict confidentiality in a secured digital environment. Samples will not be transferred to third parties and will be used in future studies on the diagnosis and prevention of HPV infections and related diseases which have been approved by CRE-HUB. Use of samples for purposes unrelated to those specified in this section will require express reconsent from study subjects.

## Discussion

Given cervical cancer is the fourth most common cancer among women worldwide, causing over 600,000 new cases and 340,000 deaths annually, two major cancer prevention strategies have been developed: primary prevention through prophylactic HPV vaccination, and secondary prevention based on the detection of HPV infections and treatable pre-cancers and early cancers [2,7]. HPV vaccines have not only proved high efficacy in the immunized but have also shown a strong herd effect [38–42]. In fact, girls-only vaccination campaigns have achieved a strong reduction of genital warts in non-vaccinated males from the same age range [40–44]. This phenomenon has been first observed with HPV vaccination given it is the only vaccine solely against a sexually-transmitted infection. These findings highlight the relevance of viral transmission and its impact in the worldwide HPV prevalence amongst sexually active individuals.

The RIFT trial aims to evaluate if a 3-dose regimen of Gardasil-9 could reduce the infective capacity of body fluids from HPV-positive women. This is based on the hypothesis that vaccine-induced antibodies, present in the epithelial mucosa, could neutralize the virions released from infected cells and prevent HPV transmission to sexual partners. In addition, we will also assess if similar outcomes can be observed using a 1- or 2-dose vaccination schedule. Demonstrating that vaccination of HPV carriers can reduce or mitigate transmission would sustain a new indication for vaccination (i.e., all HPV+ women found at routine screening) and a boost to reducing viral circulation. For that matter, exploratory results in that direction would help the rationale for larger trials that could be embedded in the context of routine screening programs that already switched to HPV testing. Furthermore, offering vaccination to HPV carriers at the time of HPV screening may also contribute to the management of these women, that should perceive vaccination as an option to reduce the risk of transmission to her current or future sexual partners.

We anticipate a few limitations in the conduct of this study. One of the setbacks can be that given there is a high percentage of transient HPV infections, which can be naturally cleared, a significant proportion of derived subjects will no longer be HPV positive by the start or during the vaccination schedule. This will result in high exclusion rates, but it has been taken into consideration when designing the study. Another limitation to be considered is that we will be able to measure humoral immunity triggered by vaccination, but we are not taking into account cell-mediated immunity, which could potentially play a role in infectivity reduction. Our dissemination plan includes the communication of obtained results in national and international congresses specialized in HPV pathology and to the general population through institutional communication channels.

Taken altogether, the findings of this study will determine if Gardasil-9 could be considered as a new complementary strategy to prevent HPV-associated diseases, by reducing viral spread. This trial could open the doors for future and bigger clinical trials involving both women and men, in order to generate scientific evidence that supports vaccination of HPV-positive adults.

## Supporting information

S1 File SPIRIT checklist. (PDF)

S2 File Clinical study protocol. (PDF)

S3 File Study questionnaire. (PDF)
