# Supplementary material for: Assessing the reduction of viral infectivity in HPV16/18-positive women after one, two, and three doses of Gardasil-9 (RIFT): Study protocol
Source: PLoS One. 2024 May 20;19(5):e0304080. doi: 10.1371/journal.pone.0304080 (PMC11104652; doi:10.1371/journal.pone.0304080)
Supplement: S2 File — (PDF) [file pone.0304080.s002.pdf]

Title Page

Protocol Title

A Non-Randomized, Open-Label Study to Assess the Reduction of Human Papillomavirus (HPV) Viral Infectivity and Transmission in HPV16/18-Positive Women Before and After Vaccination with 9vHPV, a Multivalent L1 Virus-like Particle Vaccine, Evaluated in Cervical, Anal, Vulvar, Urine and Oral Samples Obtained After One, Two, and Three Vaccine Doses (RIFT-HPV1/RIFT-HPV2).

Short Title

A Non-Randomized, Open-Label Study to Assess the Reduction of Human Papillomavirus (HPV) Viral Infectivity and Transmission in HPV-Positive Women After Vaccination with 9vHPV.

Acronym

RIFT-HPV

EudraCT Number

2021-005229-26

Document History

Document	Date	Description
Original Protocol: RIFT-HPV V3.9-00	11/11/2021	Original Protocol

PROTOCOL SUMMARY

1.1 SYNOPSIS

1.1.1 Protocol Title

A Non-Randomized, Open-Label Study to Assess the Reduction of Human Papillomavirus (HPV) Viral Infectivity and Transmission in HPV16/18-Positive Women Before and After Vaccination with 9vHPV, a Multivalent L1 Virus-like Particle Vaccine, Evaluated in Cervical, Anal, Urine, Vulvar and Oral Samples Obtained After One, Two, and Three Vaccine Doses (RIFT-HPV1/RIFT-HPV2).

1.1.2 Short Title

A Non-Randomized, Open-Label Study to Assess the Reduction of Human Papillomavirus (HPV) Viral Infectivity and Transmission in HPV-Positive Women After Vaccination with 9vHPV.

1.1.3 Acronym

RIFT-HPV

1.1.4 EudraCT Number

2021-005229-26

1.1.5 Hypotheses, Objectives, Endpoints

Objectives	Endpoints
1) **Primary Objective.** To demonstrate that vaccination with a 3-dose regimen of 9vHPV will reduce viral infectivity in cervical, anal, urine, vulvar and oral samples from HPV 16/18/16+18-positive women.<br><br>**Hypothesis.** 9vHPV promotes the production and exudation of anti-HPV antibodies, which neutralize new HPV16/18 virions and reduce the infective capacity of body fluids in HPV-positive women and the transmission of HPV infection.	• **In-vitro infectivity evaluation** (by expression of E1^E4 HPV biomarker in HaCaT keratinocytes) of cervical, anal, urine, vulvar and oral samples collected before and after 9vHPV vaccination.<br>• **Detection of HPV 6/11/16/18/31/33/45/52/58 L1 antibodies** in cervical, anal, urine, vulvar and oral samples collected before and after 9vHPV vaccination, using<br>– ELISA for types 16 and 18 in samples from RIFT-HPV 1 and 2 study cohorts,<br>– cLIA for all 9vHPV-covered types in samples from RIFT-HPV 1 study cohort.<br><br>This endpoint will allow associating the reduction in viral infectivity with the presence of neutralizing antibodies.<br><br>• **HPV16/18 virion detection** (using ELISA, electronic microscopy) **and HPV DNA**

	**detection and genotyping** (using Anyplex HPV28) in cervical, anal, urine, vulvar and oral samples collected before and after 9vHPV vaccination.<br><br>This endpoint will allow to identify samples of subjects with a non-productive viral infection or undergoing natural clearance and distinguish them from samples of subjects with productive but reduced infection due to 9vHPV vaccination.
2) **Secondary Objective.** To determine HPV antibody levels before and after vaccination (GMT and seroconversion percentages) for each of the 9vHPV-covered HPV types (6, 11, 16, 18, 31, 33, 45, 52, and 58)<br><br>**Hypothesis.** A higher level of anti-HPV antibodies is expected after 9vHPV vaccination than after a natural serological response to an HPV infection. Anti-HPV antibodies titration will allow identifying subjects with natural serological responses before vaccination.	**HPV 6/11/16/18/31/33/45/52/58 L1 antibody titration** in serum samples collected before and after 9vHPV vaccination, using<br>- ELISA for types 16 and 18 in samples from RIFT-HPV 1 and 2 study cohorts,<br>- cLIA for all 9vHPV-covered types in samples from RIFT-HPV1 study cohort.
3) **Tertiary/Exploratory Objective.** To demonstrate viral infectivity reduction in cervical, oral, urine, vulvar and anal samples from HPV 16/18/16+18-positive women before and after vaccination with 1-dose or 2-dose regimen of 9vHPV.<br><br>**Hypothesis**. Since serological response after administration of 2 vaccine doses is not inferior to 3 doses, infectivity reduction in cervical, anal, urine, vulvar and oral samples are expected to be detected after 2 doses, and at least partially after one dose.	Same endpoints as in primary objective.

1.1.6 Study Population

A minimum of 39 and 30 subjects will be included in the RIFT-HPV 1 and 2 study cohorts respectively, according to inclusion and exclusion criteria described in section 5.

All study activities listed in section 1.2.2 will be performed on RIFT-HPV 1 and 2 cohorts.

1.2 STUDY DESIGN

1.2.1 Overall Design

Primary Purpose	Prevention of HPV transmission.
Study population	RIFT-HPV 1 cohort. Non-vaccinated adult women aged 18 years or older, positive for HPV 16 and/or HPV 18 in cervical test, attending routine cervical cancer screening or the Gynaecology unit.<br><br>RIFT-HPV 2 cohort. Non-vaccinated adult women aged 18 years or older, positive for HPV 16 and/or HPV 18 in anal test ; or positive for HPV 16 and/or HPV 18 in cervical test with vulvar premalignant lesion or condylomas, associated to HPV infection, attending the infection disease or Gynaecology units.
Study Type	Interventional.
Control Type	None.
Randomization	None.
Experimental arms	Single arm.
Study Intervention	Administration of 9vHPV (Gardasil-9™) vaccine under therapeutic indication (3 doses).
Sample Collection	Collection of blood, cervical, vulvar and anal samples under clinical standard procedures, frequently used in gynecologic practice.<br><br>Collection of urine and oral samples. Although not included in the gynecologic practice, it is a simple, non-invasive and innocuous procedure, usually well-tolerated by patients.

Laboratory Procedures	Analysis of E1^E4 HPV biomarker expression in HaCaT keratinocytes in-vitro after exposure to cervical, anal, urine, vulvar or oral samples.<br><br>Detection of HPV16/18 in cervical, anal, urine, vulvar and oral samples, using ELISA/electron microscopy and HPV test/genotyping (Anyplex HPV28).<br><br>Detection and titration of anti-HPV 6/11/16/18/31/33/45/52/58 L1 antibodies in serum, cervical, anal, urine, vulvar and oral samples, using ELISA and cLIA.
Estimated Duration of Study	2 years from the time the first subject signs the informed consent until the last subject's last study visit.

1.2.2 Schedule of Activities (SoA)

	Day 1	Month 2<br>(±3 weeks)	Month 6<br>(±4 weeks)	Month 7<br>(-3 weeks/ +7 weeks from Visit 3)
	Visit 1	Visit 2	Visit 3	Visit 4
Sign Informed Consent	✓			
Review eligibility criteria	✓			
Urine sample and pregnancy test performance	✓	✓	✓	✓
Temperature (fever≥37.8°C)	✓	✓	✓	✓
Questionnaire	✓	✓	✓	✓
Height and weight measurement	✓			

Medical history, past and current medication and vaccination	✓	✓	✓	✓
Gynecological examination in routine visit	✓	✓	✓	✓
Cervical sample collection	✓	✓	✓	✓
Vulvar sample collection	✓	✓	✓	✓
Anal sample collection	✓	✓	✓	✓
First-void urine collection	✓	✓	✓	✓
Oral sample collection	✓	✓	✓	✓
Blood sample collection	✓	✓	✓	✓
Vaccination + 30 min monitoring for post-vaccination immediate adverse events	✓	✓	✓	
AE/SAE assessment		✓	✓	✓
Laboratory analytical procedures				
HPV infective capacity in-vitro evaluation (E1^E4-HaCaT model)	✓	✓	✓	✓
Anti-HPV 6/11/31/33/45/52/58 L1 ab detection/titration by cLIA in cervical, anal, oral, urine, vulvar and blood samples from RIFT/HPV 1 cohort	✓	✓	✓	✓
Anti-HPV 16/18 L1 ab detection/titration by ELISA in cervical, anal, oral, urine, vulvar and blood samples from RIFT/HPV 1 and 2 cohorts	✓	✓	✓	✓
HPV test and genotyping (Anyplex HPV28) in cervical, anal, urine, vulvar and oral samples	✓	✓	✓	✓

HPV16/18 virion detection (ELISA, electronic microscopy) in cervical, anal, urine, vulvar and oral samples	✓	✓	✓	✓

1.3 STUDY SPONSOR, SITES, PERSONNEL

STUDY SPONSOR INVESTIGATOR	Miquel Àngel Pavón Ribas.<br><br>Cancer Epidemiology Research Program (PREC), Catalan Institute of Oncology (ICO-Hospitalet)/ Bellvitge Biomedical Research Institute (IDIBELL), Barcelona, Spain.<br><br>mpavon@iconcologia.net<br><br>93 260 71 23
STUDY SITE	Gynaecology Unit, Bellvitge University Hospital (HUB), Barcelona, Spain.
STUDY INSTITUTIONAL REVIEW BOARD/ INDEPENDENT ETHICS COMMITTEE	Bellvitge University Hospital (HUB) Research Ethics Committee.
STUDY SATELLITE SITES	Oncologic Gynaecology Unit, Hospital del Mar-Mar Health Park, Barcelona, Spain.<br><br>Investigator:<br><br>Josep Maria Solé Sedeño.<br>josepm.sole@gmail.com
	Sexual and Reproductive Health Care Centre-ASSIR Delta del Llobregat, Barcelona, Spain.<br><br>Investigators:<br><br>Clara Grau Bravo<br>cgrau.apms.ics@gencat.cat<br><br>Blas Rupérez Pérez<br>bruperez.apms.ics@gencat.cat
	Infections Unit, Bellvitge University Hospital (HUB), Barcelona, Spain.

	Investigator:<br>Maria Saumoy Linares.<br>msaumoy@bellvitgehospital.cat<br>Mónica Sánchez Llamas<br>monicasl@bellvitgehospital.cat
	The Cervical Cancer Screening Technical Office as part of Cervical Cancer Early Detection Programme of Cancer Epidemiology Research Programme of Catalan Institute of Oncology.<br>Paula Peremiquel Trillas<br>paula.peremiquel@iconcologia.net
STUDY COORDINATOR INVESTIGATORS	Francesc Xavier Bosch José.<br>Cancer Epidemiology Research Program (PREC), Catalan Institute of Oncology (ICO-Hospitalet)/ Bellvitge Biomedical Research Institute (IDIBELL), Barcelona, Spain<br>xbosch@idibell.cat
	Miguel Àngel Pavón Ribas.<br>Cancer Epidemiology Research Program (PREC), Catalan Institute of Oncology (ICO-Hospitalet)/ Bellvitge Biomedical Research Institute (IDIBELL), Barcelona, Spain.<br>mpavon@iconcologia.net
STUDY INVESTIGATORS	PRINCIPAL INVESTIGATOR<br>Miguel Àngel Pavón Ribas.<br>Cancer Epidemiology Research Program (PREC), Catalan Institute of Oncology (ICO-Hospitalet)/ Bellvitge Biomedical Research Institute (IDIBELL), Barcelona, Spain.<br>mpavon@iconcologia.net
	SUB-INVESTIGATOR

	Carlos Ortega Expósito<br>Gynaecology Unit, Bellvitge University Hospital (HUB), Barcelona, Spain.<br>cortegae@bellvitgehospital.cat
	SUB-INVESTIGATOR<br>Juan Carlos Torrejón Becerra<br>Gynaecology Unit, Bellvitge University Hospital (HUB), Barcelona, Spain.<br>jtorrejon@bellvitgehospital.cat
	SUB-INVESTIGATOR<br>Maria Eulàlia Fernández Montolí<br>Gynaecology Unit, Bellvitge University Hospital (HUB), Barcelona, Spain.<br>mfernandez@bellvitgehospital.cat
	SUB-INVESTIGATOR<br>Álvaro de Andrés Pablo.<br>Cancer Epidemiology Research Program (PREC), Catalan Institute of Oncology (ICO-Hospitalet)/ Bellvitge Biomedical Research Institute (IDIBELL), Barcelona, Spain.<br>adeandres@idibell.cat
	SUB-INVESTIGATOR<br>Victòria López Codony<br>Cancer Epidemiology Research Program (PREC), Catalan Institute of Oncology (ICO Hospitalet)/Bellvitge Biomedical Research Institute (IDIBELL), Barcelona, Spain.<br>vlopez@idibell.cat

STUDY NURSE	Yolanda Pérez Escanilla.<br><br>Gynaecology Unit, Bellvitge University Hospital (HUB), Barcelona, Spain.).<br><br>yperez@bellvitgehospital.cat
STUDY PHARMACIST	Anna Maria Ferrer Artola.<br><br>Pharmacy Unit, Bellvitge University Hospital (HUB), Barcelona, Spain.<br><br>aferrer@bellvitgehospital.cat
STUDY STATISTICIAN	Sara Tous Belmonte<br><br>Cancer Epidemiology Research Program (PREC), Catalan Institute of Oncology (ICO-Hospitalet)/ Bellvitge Biomedical Research Institute (IDIBELL), Barcelona, Spain.<br><br>stous@iconcologia.net
STUDY COORDINATOR	Marta López Querol.<br><br>Cancer Epidemiology Research Program (PREC), Catalan Institute of Oncology (ICO-Hospitalet)/ Bellvitge Biomedical Research Institute (IDIBELL), Barcelona, Spain.<br><br>mlopezq@idibell.cat
VACCINE MANUFACTURER AND DISTRIBUTOR	Manufacturer: Merck Sharp & Dohme (MSD)<br><br>Distributor: Alcura
LABORATORY ASSESSMENTS	Cancer Epidemiology Research Program (PREC), Catalan Institute of Oncology (ICO-Hospitalet)/ Bellvitge Biomedical Research Institute (IDIBELL), Barcelona, Spain.<br><br>- HPV viral infectivity<br>- HPV16/18 virion detection using ELISA, electronic microscopy<br>- HPV DNA detection and genotyping using Anyplex HPV28

	- Anti-HPV antibodies detection (HPV16/18) using ELISA.<br>MSD through designated vendor:<br>- Anti-HPV antibodies detection and titration using cLIA
STUDY CRO	Clinical Research and Clinical trial Unit (UICEC-IDIBELL), Bellvitge University Hospital (HUB)<br>ucicecidibell@bellvitgehospital.cat<br>93 260 7107<br>- Regulatory proceedings<br>- Study's external monitoring

2 INTRODUCTION

2.1 Study Rationale

By excluding adult-women and men from vaccination, the population's vaccination coverage and the herd protection effect are considerably reduced. Individuals with a productive infection can be an important "silent" reservoir of HPV, thereby facilitating the perpetuation of HPV through its transmission to new individuals. Considering the presence of viral particles and anti-L1 antibodies in cervical, anal, urine, vulvar and oral fluids from vaccinated and HPV-positive individuals, and the reduction of certain HPV types in male partners of women that have been vaccinated, the primary objective of this study is based on the hypothesis that neutralizing antibodies (nAbs) transudated in the epithelial mucosa could neutralize HPV viral particles released from infected cells and inhibit transmission of the infection and the activation of a new viral cycle. This hypothesis suggests that vaccination with 9vHPV in HPV-positive women could be a good approach to break the viral infection chain between individuals. To demonstrate this hypothesis, the study is designed to evaluate HPV infective capacity of body fluids (cervical, anal, urine, vulvar and oral samples), the seroconversion status, and the presence of HPV antibodies and viral particles in adult HPV-positive women before and after vaccination with the standard schedule of three doses of 9vHPV.

The E1^E4-HaCaT model allows to evaluate, before and after vaccination, if viral particles present in body fluids of HPV-positive individuals are functional or have been neutralized through pre- or post-entry mechanisms which avoid the activation of the viral cycle. E1^E4 protein RNA will be a surrogated marker to evaluate the number of keratinocytes that have been infected after incubation with body fluids containing viral particles. The detection of anti-HPV antibodies in serum before and after vaccination will allow to identify individuals showing natural serological responses, which will be enhanced after vaccination.

9vHPV will not have any effect on the elimination of the HPV infection already present or the production of new viral particles. However, the detection of HPV DNA and virions before and after vaccination is essential to identify women showing spontaneous viral clearance or latent or non-productive infection and distinguish these effects from the infectivity neutralization induced after vaccination. Differences associated with anatomical location, viral exposition, antibody availably and viral replication are characteristics that can be reflected in the analysis of cervical, anal, urine, vulvar and oral samples.

The proof of concept developed in this study is essential to generate scientific evidence that endorses vaccination in adult individuals. Although this study is focused on HPV-positive women, laboratory working methods and results obtained in oral and anal samples could be the basis for a future study on HPV-positive men, also a silent reservoir for HPV infection. Results may be not only determinant in designing more effective population-based vaccination programs following the 2018 World Health Organization recommendations for cervical cancer elimination, but also have an impact on the incidence of non-cervical anogenital and oropharyngeal cancers in both men and women.

2.2 Background

2.2.1 HPV epidemiology

At least 610,000 cancer cases are attributed annually to high-risk Human Papillomavirus (HPV) infections, including all cervical cancers and a significant proportion of ano-genital (vulvar, vagina, penile, and anal) and oropharyngeal cancers[1,2]. Cervical cancer affects women in the prime of life, at a time when they are raising and caring for families and contributing socially and economically to their communities, therefore the development of new strategies for cervical cancer prevention is undoubtedly a priority area for the improvement of women's health that could generate a strong economic and social impact on the world population.

Understanding the role of HPV in cervical cancer (the third most common cancer in women worldwide, producing more than 310.000 annual deaths) has promoted the development of two major cancer prevention strategies: primary prevention through prophylactic HPV vaccination, and secondary prevention based on the detection of HPV infection and treatable pre-cancers and early cancers[1,2].

On May 19th, 2018 the World Health Organization's Director-General made a global call for action towards the elimination of cervical cancer[3]. In response to this call, the governments of many countries are now interested in identifying the best strategy to combine vaccination and screening and obtain the best cost-benefit balance.

2.2.2 HPV viral cycle

More than 200 human papillomaviruses have been described until now, but only a limited group of 12 genotypes (16, 18, 31, 33, 35, 39, 45, 51, 52, 56, 58 and 59), showing carcinogenic capacity, have been classified by the IARC as high-risk HPV types (HR-HPV)[4]. HPV16 is the most carcinogenic type and is detected in more than 70% of cervical cancers. Despite being a heterogeneous group of viruses, the genome structure and associated carcinogenic process for all HR-HPV is similar. A circular double-stranded DNA genome encodes the capsid proteins (L1 and L2), which are crucial for the first steps of binding and viral entry and are also important to

generate a type-specific immune response. Initial HPV16 binding occurs via heparan-sulphate proteoglycans (HSPGs) located on the epithelial cells[5,6]. Cyclophilin B mediates the conformational change in the virus capsid that increases the exposure of the N-terminus of L2 protein and facilitates efficient infection. HPV16 genome also encodes a group of early proteins named E1, E2, E4, E5, E6, and E7. E1, E2 and E4 are essential to maintain the viral fitness (replication, virion synthesis and release). E1^E4 fusion protein is the most highly expressed protein in the productive life cycle of human papillomaviruses; it contributes to multiple facets of the virus life cycle and may facilitate efficient virus release and transmission[7]. E6 and E7 (and partially E5) are mainly involved in the carcinogenic process[8].

2.2.3 HPV prophylactic vaccines, viral entry and antibody neutralization

Prophylactic vaccines are based on the expression of recombinant L1 protein subunits that can self-assemble generating virus-like particles (VLPs), with type-specific conformation, which mimics morphological and immunological characteristics of native virions[8]. L1 VPLs-based vaccines have demonstrated a high degree of safety, immunogenicity and effectiveness, preventing HPV infection and malignant lesions caused by the targeted genotypes.

9vHPV (Gardasil-9™, manufactured by MSD) protects against 9 genotypes (HPV 6, 11, 16, 18, 31, 33, 45, 52, and 58) that together are responsible for 90% of cervical cancer cases. It was originally licensed as a 3-dose regimen, which is the regimen adopted for this study. It contains amorphous aluminium hydroxyphosphate sulphate as adjuvant[9].

Although the mechanism of protection is not fully understood, is largely accepted that neutralizing antibodies (nAbs) play a crucial role preventing virus particle binding to the epithelial cell surface and inhibiting viral entry. The available data suggest that nAbs can block early extracellular events preventing the interaction between L1 and heparin sulphate proteoglycans on the basement membrane. nAbs can also block those binding effects that occur before and after changes in L2 protein[10,11]. An alternative post-entry nAbs mechanism that involves the activation of TRIM21 pathway has been described in a wide range of non-enveloped virus. TRIM21, which resides in the cytoplasm, targets the viral nAbs- labelled particles for proteasomal degradation after completing the entry[12].

Regardless of the specific mechanism of neutralization, nAbs produced after vaccination confer a long period of individual protection against a new HPV infection, which is much more effective than a natural serological response. HPV vaccines have shown high efficacy, immunogenicity, and safety also in individuals previously exposed to HPV[13]. However, L1-VLP vaccines are prophylactic, not therapeutic: once the HPV infection is present, nAbs generated in response to vaccination can prevent new infections, but cannot eliminate the virus-infected cells. It is for this reason that most HPV vaccination programs are aimed at adolescents before the first sexual encounter occurs [1]. Little is known about the effect of vaccination in the transmission of HPV infection between sexual partners.

2.2.4 Antibody and viral particle detection in body fluids and models to assess viral infectivity

Previous studies have shown that anti-L1 antibodies are present in cervicovaginal and oral fluids in vaccinated women, in asymptomatic men or women with a positive serology for HPV16, and in oral mucosal transudates from HIV/HPV-positive individuals[13–22,57].

Vaccine-induced HPV antibodies originating from cervicovaginal secretions have also recently detected in first-void (FV) urine[52]. Discharged mucus and debris from exfoliated cells from the female genital organs accumulate around the urethra opening, between the small labia, and are washed away mainly with the urine initial flow (first-void urine)[53].

Moreover, new viral particles are present in body fluids obtained from individuals showing a productive HPV infection[23,24] and HPV DNA has been detected in first-void urine[54]. Although the direct interaction between antibodies and viral particles has not been demonstrated so far in the same group of HPV-positive individuals, the simultaneous detection of viral particles and anti-L1 antibodies in cervical, anal, vulvar and oral fluids suggest that anti-L1 antibodies, despite being unable to suppress the production and release of new viral particles, could decorate the viral capsid and limit their infectivity. As the vaccine VLPs induce a higher antibody response compared to natural serological responses, it could be expected that the interaction between anti-L1 antibodies and new produced viral particles, and therefore, viral neutralization, will be more effective in vaccinated women. In line with this, an evaluation of HPV transmission among heterosexual couples in the HITCH prospective cohort yielded a reduced transmission associated to vaccinated women[25], and a currently ongoing clinical trial is evaluating the effect of vaccination on the reduction of HPV DNA positivity incidence in sexually active couples (NCT01824537)[26,27]. However, new assays to functionally assess the reduction of infective capacity are indispensable to distinguish the effect of the vaccine on the prevention of new infections, from the effect on the infective capacity and transmission of HPV infections already present before vaccination.

Serology techniques based on neutralization assays, competitive immunoassays and ELISA have been used to evaluate antibody response to HPV vaccines[17]. Although neutralization assays mimic functions closest to the natural mechanisms, they require cell culture and are time-consuming. ELISA assays are fast and easy to multiplex, but they detect neutralizing and non-neutralizing antibodies. In HPV-positive, individuals that have been vaccinated (immunocompromised individuals or post-treatment vaccination) serology assays are unable to distinguish antibodies produced as a natural response to viral particles from the antibodies induced after vaccination.

Because HPV life cycle is dependent on the differentiation of basal cells into keratinocytes, it is challenging to study in vitro[28]. 3D organotypic raft culture systems that mimic epithelial cell differentiation in vitro have been used to produce virions with infectious potential. However, 3D cultures are usually complex to standardize and replicate, what make them inefficient for large-scale epidemiologic studies or clinical trials. Although HPV cannot complete the viral cycle in a 2D cell culture, 2D models are easy to standardize and have emerged as useful tools for analyzing the first steps associated with viral binding, cell-entry and infection[29]. Ozbun and co-workers developed a model to analyze the HPV infective capacity based on the culture of HaCaT

keratinocytes[30]. HaCaT cells are incubated with biological samples potentially containing viral particles and after 24-48 hours the expression of the E1^E4 mRNA is used to quantify the number of cells that have been infected. E1^E4 mRNA expression is essential to maintain viral fitness and complete the viral cycle and is used as a surrogated marker that indicates infection of epithelial cells and activation of the viral replication machinery. Differences in E1^E4 activity impacts several processes such as viral capsid assembly, maturation and infectivity[31]. This functional assay can quantify infectious particles present in the sample, through the quantification of cells expressing E1^E4 mRNA. In the present study, we will evaluate two different protocols (see section 8.2.2) to analyze the expression on E1^E4 mRNA in HaCaT cells exposed to cervical, anal, urine, vulvar or oral samples with the aim of developing a standardized and reproducible protocol for the development of larger clinical trials in women and men.

2.2.5 Pharmaceutical and Therapeutic Background

The 9vHPV vaccine (Gardasil-9™) is an aluminium-adjuvanted recombinant protein vaccine prepared from the highly purified VLPs of the recombinant major capsid (L1) protein of HPV Types 6, 11, 16, 18, 31, 33, 45, 52, and 58.

The 9vHPV vaccine is currently indicated by the European Medicines Agency (EMA) in males and females from the age of nine years for the prevention of dysplastic lesions and cancers of the cervix, vagina, vulva, and anus, as well as for prevention of genital warts caused by the HPV types targeted by the vaccine[9].

According to the US Food and Drug Administration (FDA), the 9vHPV vaccine is indicated in males and females 9 through 45 years of age for the prevention of the following diseases caused by the vaccine-targeted HPV types: intraepithelial neoplasia of the cervix, vagina, vulva and anus, genital warts and cervical adenocarcinoma in situ. In males 9 through 45 years of age, it is also indicated for the prevention of anal, oropharyngeal and other head and neck cancers caused by the vaccine-targeted HPV types[32].

2.2.6 Clinical and Population-Based Studies

Safety of HPV vaccines has been ascertained through evaluation of surveillance data obtained from more than 270 million doses administered in immunization programs, showing no association with serious adverse events[33,34].

Efficacy of HPV vaccines in clinical trials is ~100% in a three-dose regimen in HPV-negative women younger than 25 years of age, for protection against cervical persistent infection and cervical pre-cancerous lesions, and sustained protection has been demonstrated for up to 12 years of follow-up in several population-based studies[33,34]. Protection against cervical pre-cancerous lesions in older women (24 to 45 years) has also been demonstrated, although efficacy is lower compared to younger women[34,35].

Results from a recent population-based study in Sweden showed a ~90% reduction in cervical cancer incidence in vaccinated women younger than 17 years of age[36]. The same study showed a lower but robust reduction (53%) in cervical cancer incidence in women vaccinated at 17 to 30 years of age.

A recent meta-analysis including randomized clinical trials and several retrospective, prospective and cancer registry studies showed a significant risk reduction of new cervical pre-cancerous lesions after cervical excision in women vaccinated at a time close to surgery. Results of this meta-analysis demonstrated no difference in risk reduction between women younger than 25 years and older women[37], and are thus an additional argument in favour of vaccination of HPV-positive older women.

Regarding HPV transmission reduction and prevention, analysis of data from a prospective cohort study of heterosexual couples (HITCH) yielded a reduction in HPV transmission to their male partners from vaccinated women compared to couples with unvaccinated women, and those vaccinated women with infections of HPV types covered by the vaccine (acquired prior to vaccination) had lower viral loads than unvaccinated women[25]. Also, the currently ongoing TRAP-HPV study will evaluate the effect of vaccination on the reduction of HPV DNA positivity incidence in sexually active couples (NCT01824537)[27]. This study is phase 4, interventional, randomized to receive 9vHPV vaccine or a placebo (Hepatitis A vaccine), and is expected to enroll 1000 unvaccinated subjects of 18-45 years of age[26].

2.2.7 Benefit/Risk Assessment

The 9vHPV vaccine has been shown to be beneficial and efficacious in preventing persistent anogenital HPV infection and disease associated with the 9 HPV types (6, 11, 16, 18, 31, 33, 45, 52, and 58). The frequency, severity, and magnitude of AEs identified in previous studies and post-marketing surveillance support a favorable benefit-risk analysis for the 9vHPVvaccine in the study population. In this study, the 9vHPV vaccine will be administered in the same 3-dose regime as currently used for the vaccination of girls through the Spanish National HPV Immunization Program[38].

Study subjects will benefit from participating in the study by receiving the 9vHPV vaccine, which is currently not included in the Spanish National Immunization Program for adult women[38]. Although vaccination is unable to eliminate HPV infections already present, most HPV infections are cleared after 12-24 months, thus vaccination with 9vHPV will confer protection for future HPV persistent infections in women enrolled in the study.

Study subjects will also benefit from a gynecological examination by trained and experienced medical personnel, who may refer her for appropriate care and treatment.

3 HYPOTHESES, OBJECTIVES AND ENDPOINTS

Objectives	Endpoints
1) **Primary Objective.** To demonstrate that vaccination with a 3-dose regimen of 9vHPV will reduce viral infectivity in cervical, anal, urine, vulvar and oral samples from HPV 16/18/16+18-positive women.	• **In-vitro infectivity evaluation** (by expression of E1^E4 HPV biomarker in HaCaT keratinocytes) of cervical, anal, urine, vulvar and oral samples collected before and after 9vHPV vaccination.

**Hypothesis.** 9vHPV promotes the production and exudation of anti-HPV antibodies, which neutralize new HPV16/18 virions and reduce the infective capacity of body fluids in HPV-positive women and the transmission of HPV infection.	• **Detection of HPV 6/11/16/18/31/33/45/52/58 L1 antibodies** in cervical, anal, urine, vulvar and oral samples collected before and after 9vHPV vaccination, using<br>– ELISA for types 16 and 18 in samples from RIFT-HPV 1 and 2 study cohorts,<br>– cLIA for all 9vHPV-covered types samples from RIFT-HPV 1 study cohort.<br><br>This endpoint will allow to associate the reduction in viral infectivity with the presence of neutralizing antibodies.<br><br>• **HPV16/18 virion detection** (using ELISA, electronic microscopy) **and HPV DNA detection and genotyping** (using Anyplex HPV28) in cervical, anal, urine, vulvar and oral samples collected before and after 9vHPV vaccination.<br><br>This endpoint will allow to identify samples of subjects with a non-productive viral infection or undergoing natural clearance, and distinguish them from samples of subjects with productive but reduced infection due to 9vHPV vaccination.
2) **Secondary Objective.** To summarize antibody responses to vaccination (GMT and seroconversion percentages) for each of the 9vHPV-covered HPV types (6, 11, 16, 18, 31, 33, 45, 52, and 58)<br><br>**Hypothesis.** A higher level of anti-HPV antibodies is expected after 9vHPV vaccination than after a natural serological response to an HPV infection. Anti-HPV antibodies titration will allow to identify subjects with natural serological responses before vaccination.	• **HPV 6/11/16/18/31/33/45/52/58 L1 antibody titration** in serum samples collected before and after 9vHPV vaccination, using<br>– ELISA for types 16 and 18 in samples from RIFT-HPV 1 and 2 study cohorts,<br>– cLIA for all 9vHPV-covered types in samples from RIFT-HPV 1 study cohort.
3) **Tertiary/ Exploratory Objective.** To demonstrate viral infectivity reduction in cervical, oral, urine, vulvar and anal samples from HPV 16/18/16+18-positive	• Same endpoints as in primary objective.

women before and after vaccination with 1-dose or 2-dose regimen of 9vHPV.<br>**Hypothesis**. Since serological response after administration of 2 vaccine doses is not inferior to 3 doses, infectivity reduction in cervical, anal, vulvar, urine and oral samples is expected to be detected after 2 doses, and at least partially after one dose.	

4 STUDY DESIGN

4.1 OVERALL DESIGN

The main objective of the study is to demonstrate the reduction in infective capacity of body fluids (cervical, anal, urine, vulvar and oral samples) in adult women at least HPV 16 and/or 18 positive after vaccination with 9vHPV in a three-dose regimen.

The study's secondary/ tertiary objectives are the following:

- To determine HPV antibody levels before and after vaccination (GMT and seroconversion percentages) for each of the 9vHPV-covered HPV types (6, 11, 16, 18, 31, 33, 45, 52, and 58).
- To demonstrate viral infectivity reduction in cervical, oral, urine, vulvar and anal samples from women positive for at least HPV 16 and/or 18 HPV before and after vaccination with 1-dose or 2-dose regimen of 9vHPV.

A minimum of 39 and 30 subjects will enroll in two different study population cohorts, respectively:

RIFT-HPV 1 cohort:

- Non-vaccinated adult women aged 18 years or older, positive on cervix for HPV 16 and/or 18 with non-apparent cervical lesion or with cervical intraepithelial neoplasia (CIN) 1/2 lesion on cytology or biopsy, eligible for **conservative treatment**.

RIFT-HPV 2 cohort:

- Non-vaccinated adult women aged 18 years or older, positive for HPV 16 and/or HPV 18 anal test with non-apparent anal lesions or with anal lesions eligible for **conservative treatment**.
- Or non-vaccinated adult women aged 18 years or older, positive for HPV 16 and/or HPV 18 cervical test with vulvar premalignant lesion or condylomas, associated to HPV infection**.**

Candidates will be selected to participate according to the HPV DNA test result in a sample taken in their routine gynecological follow-up visit in:

- The Gynaecology Unit of the Bellvitge's University Hospital, which is designated as the study site, and where all study procedures are to be performed by the Sub-investigator and Study Nurse.
- those sites designated as satellite, which will contribute to the study only in subject's refer to study site to evaluate her enrolment:
    - The Oncologic Gynaecology Unit of the Mar Health Park (Study Investigator: Josep Maria Solé Sedeño).
    - The Sexual and Reproductive Health Care Centre-ASSIR Delta del Llobregat (Study Investigators: Clara Grau Bravo and Blas Rupérez Pérez).
    - The Infections Unit of the Bellvitge's University Hospital (Study Investigators: Maria Saumoy Linares, Mónica Sánchez).
    - the Cervical Cancer Screening Technical Office as part of Cervical Cancer Early Detection Programme of Cancer Epidemiology Research Programme of Catalan Institute of Oncology (Study Investigator: Paula Peremiquel Trillas)
- The patients know the Study through our spreading of information material (mailing, posters, flyers,…) and they contact to Study team.

Information of study Investigators and sites is detailed in section 1.3.

Those women with a at least HPV 16 and/or 18-positive result and matching the baseline characteristics defined for RIFT-HPV 1 or 2 study cohorts will be invited to participate in the study by the Principal Investigator in the study site or the Investigators in the study's satellite sites. After explaining the objectives and procedures of the study to pre-selected candidates and answer their questions, Investigators of satellite sites will offer them to sign a Personal Data Usage Authorization Form (annex 11.3) where they agree to be contacted by the study coordinator to make an appointment for the study's Visit 1 in the study site.

During Visit 1, eligibility criteria defined in section 5 will be reviewed with subjects after they read the Subject's Information Sheet and sign the Informed Consent Form (annex 11.2). Those subjects complying with inclusion and exclusion criteria will be enrolled in the study and included in RIFT-HPV 1 or 2 study cohorts according to baseline characteristics.

All enrolled subjects will complete the specified procedures in 4 study visits: Visit 1/Day 1, Visit 2/Month 2, Visit 3/Month 6, and Visit 4/Month 7. Study procedures and time windows for each visit are summarized in the Schedule of Activities (section 1.2.2).

9vHPV vaccine will be administered in Visits 1, 2 and 3 by a qualified health professional (Study nurse/Sub-Investigator).

There is no control group in this study: all subjects signing the Informed Consent Form will receive 9vHPV vaccination in a three-dose regimen as per protocol (section 8.1.9) and therapeutic indication[38–40].

All enrolled subjects will follow the study procedures summarized below as per protocol (section 1.2.2):

- Pregnancy test on a urine sample in Visits 1, 2, 3 and 4 before receiving any vaccine or collecting any sample.
- Completion of a questionnaire in Visits 1 and 4.
- Cervical, vulvar and anal sample collection by the study Investigator, and oral, urine and blood sample collection by the study nurse. All samples will be collected in Visits 1, 2 and 3 before receiving 9vHPV vaccination, and in Visit 4.
- Intramuscular administration of 9vHPV in a three-dose regimen in Visits 1, 2 and 3.

The following study laboratory procedures will be applied to samples obtained from study subjects:

- Assessment of HPV infectivity. Analysis of E1^E4 HPV biomarker expression in HaCaT keratinocytes in-vitro after exposure to cervical, anal, urine, vulvar or oral samples from all study subjects.
- Assessment of HPV positivity. Detection of HPV16 and 18 in cervical, anal, urine, vulvar and oral samples from all study subjects, using ELISA/electron microscopy to detect HPV virions, and HPV test/genotyping (Anyplex HPV28) to detect HPV DNA.
- Assessment of HPV immunogenicity. Detection and titration of anti-HPV L1 antibodies in serum, cervical, anal, urine, vulvar and oral samples from all study subjects, using ELISA for types 16 and 18 in samples from RIFT-HPV 1 and 2 study cohorts, or cLIA for all 9vHPV-covered types in samples from RIFT-HPV 1 study cohort.

Study subjects will be followed thorough the whole duration of the study for possible injection-site AEs, systemic AEs, SAEs and deaths regardless of causality. All AEs and SAEs will be recorded by the study Investigator at each study visit (section 8.4).

4.1.1.1 Study procedures under therapeutic indication

The nonavalent HPV vaccine (9vHPV or Gardasil-9™) was first authorized for marketing in Europe in June 2015, and is currently indicated in individuals from 9 years of age for the prevention of diseases caused by vaccine's 9 HPV types: genital warts (HPV6 and 11) and premalignant lesions and cancers affecting the cervix, vulva, vagina and anus (HPV 16, 18, 31, 22, 45, 52 and 58)[9].

HPV vaccination is recommended by the Spanish Ministry of Health and is included in the National Immunization Program for 12 to 18-year-old girls and adult population at risk, including women treated with cervical excision with no age limit, and HIV-positive individuals under 26 years of age[38–40].

Therefore, adult women outside the risk population defined by the Ministry of Health are excluded from the National HPV Immunization Program. If eligible for inclusion, these women will benefit from participation in the present study by receiving 9vHPV vaccination free of charge.

Cervical, anal, vulvar and blood samples will be collected under standard clinical procedures, frequently used in gynecological practice (see section 8.2.1). Procedures described in this protocol are used to collect samples in the following clinical routine practice:

- Cervical samples: in women during their cervical cancer screening or follow-up visits, to perform cytology/HPV testing.

- Anal samples: in patients 40 years or older, testing positive for HPV16/18 in a cervical sample collected during a routine screening or follow-up visit.
- Vulvar samples: in women testing positive for HPV16/18 in a cervical sample collected during a routine screening or follow-up visits
- Blood samples: in patients before genital surgery or under other clinical circumstances that make it necessary by medical judgement.

Collection of oral samples does not belong to routine clinical practice. However, this and first-void urine sample are simple, non-invasive and innocuous procedures, and is generally well-tolerated by subjects (see section 8.2.1).

4.1.1.2 Statistical analysis

Study data description and analysis, as well as study's population size and statistical power are detailed in section 9.

4.2 SCIENTIFIC RATIONALE FOR STUDY DESIGN

4.2.1 Rationale for Study Population

The study population is composed of two different cohorts.

The RIFT-HPV 1 cohort. It includes non-vaccinated adult women aged 18 years or older, positive on cervix for HPV 16 and/or 18 with non-apparent cervical lesion or with cervical intraepithelial neoplasia (CIN) 1/2 lesion on cytology or biopsy, eligible for **conservative treatment.**

Eligible women for this cohort are expected to frequently have productive cervical HPV infections (i.e., with high production of virions capable of infecting new cells) that allow the evaluation of HPV infectivity in cervical samples using the E1^E4-HaCaT essay, before and after vaccination with 9vHPV in a three-dose regimen.

Women in this cohort are generally expected to have a low prevalence of anal HPV infections (5% to 22% in women with no HPV-related pathology)[41] and therefore, the obtention of anal samples with productive HPV infections is expected to be scarce.

The RIFT-HPV 2 cohort. It includes non-vaccinated adult women aged 18 years or older, positive for HPV 16 and/or HPV 18 anal test with non-apparent anal lesions or with anal lesions eligible for **conservative treatment**. Or non-vaccinated adult women aged 18 years or older, positive for HPV 16 and/or HPV 18 cervical test with vulvar premalignant lesion or condylomas, associated to HPV infection.

Eligible women for this cohort are expected to frequently have productive cervical and anal HPV infections that allow the evaluation of HPV infectivity in cervical, vulvar and anal samples using the E1^E4-HaCaT essay, before and after vaccination with 9vHPV in a three-dose regimen.

Since anal HPV infections are more prevalent in women with HPV-related cervical or vulvar/vaginal lesions (23% to 86%)[41], this second study cohort will guarantee the obtention of anal and vulvar samples with productive HPV infections.

Oral and urine samples will be obtained from study subjects belonging to both study cohorts. HPV infectivity in oral and urine samples with productive HPV infections will be also evaluated in the same conditions as cervical, vulvar and anal samples.

The analysis performed on anal, urine, vulvar and oral samples obtained from the combination of both study cohorts will increase the statistical power of the analysis performed in cervical samples. Moreover, results on the evaluation of the impact of HPV vaccination on infective capacity of the virions present in cervical, anal, urine, vulvar and oral samples may yield valuable information about HPV infectivity in different anatomical areas that are involved in the transmission process of the virus.

Therefore, this study's results can be the necessary proof-of-concept (i.e. HPV vaccination can reduce viral infectivity and prevent viral transmission) to generate scientific evidence that 1) may endorse HPV vaccination in adult women and promote the design of more effective population-based vaccination programs for preventing cervical cancer; 2) may open the door to additional studies of HPV infectivity and transmission on male and female populations, focused on the reduction of the incidence of non-cervical anogenital and oropharyngeal cancers.

The size of the study population was calculated using the primary endpoint (reduction of HPV infective capacity after 3 doses of 9vHPV) as a reference. Accepting an alpha risk of 0.05 and a beta risk of less than 0.2 in a bilateral contrast, it is estimated that a minimum of 39 and 28 subjects for the first and second study cohorts respectively should be included in the study.

4.3 VACCINE DOSE AND REGIMEN

The 0.5 mL dose of 9vHPV administered as a 3-dose regimen (Day 1, Month 2, Month 6) is based on the global efficacy, immunogenicity, and safety clinical studies that supported the licensure of the 9vHPV vaccine and is consistent with the approved dosing and product labelling of Gardasil-9™[9].

4.4 BEGINNING AND END OF STUDY DEFINITION

The overall study begins when the first subject signs the ICF. The overall study ends when the last subject completes the last study-related telephone-call or visit, withdraws from the study, or is lost to follow-up (i.e., the subject is unable to be contacted by the Investigator).

For purposes of analysis and reporting, the overall study ends when the Sponsor receives the last laboratory result or at the time of final contact with the last subject, whichever comes last.

5 STUDY POPULATION

The study population is composed of two cohorts:

RIFT-HPV 1 cohort: non-vaccinated adult women aged 18 years or older, positive on cervix for HPV 16 and/or 18 with non apparent cervical lesion or with cervical intraepithelial neoplasia (CIN) 1/2 lesion on cytology or biopsy, eligible for **conservative treatment**

RIFT-HPV 2 cohort: non-vaccinated adult women aged 18 years or older, positive for HPV 16 and/or HPV 18 anal test with non-apparent anal lesions or with anal lesions eligible for **conservative treatment**. Or non-vaccinated adult women aged 18 years or older, positive for

HPV 16 and/or HPV 18 cervical test with vulvar premalignant lesion or condylomas, associated to HPV infection**.**

5.1 INCLUSION CRITERIA

Candidates are eligible for inclusion in the study if they:

- Are women, aged 18 years or older for cohort 1 and cohort 2 , attending a routine cervical cancer screening visit or gynecological visit, are positive for HPV16 and/or HPV18 have been recently diagnosed for their HPV-positivity (within the last 10 months) and meet one of the following criteria:

  R**<u>IFT-HPV 1 cohort:</u>** non-vaccinated adult women aged 18 years or older, positive on cervix for HPV 16 and/or 18, with non apparent cervical lesion or with cervical intraepithelial neoplasia (CIN) 1/2 lesion on cytology or biopsy, eligible for **conservative treatment**.

  <u>RIFT-HPV 2 cohort</u>: non-vaccinated adult women aged 18 years or older, positive for HPV 16 and/or HPV 18 anal test with non-apparent anal lesions or with anal lesions eligible for **conservative treatment**. Or non-vaccinated adult women aged 18 years or older, positive for HPV 16 and/or HPV 18 cervical test with vulvar premalignant lesion or condylomas, associated to HPV infection**.**

- Are judged to have no major health conditions (based on medical history, physical examination, and laboratory testing) that may compromise their capacity to comply with study procedures, as per Investigator's judgement.
- Provide written informed consent for their participation in the study.
- Provide a frequent contact telephone number as well as an alternate means of contact (such as an alternate telephone number or email) for follow-up purposes.
- Are planning to stay in their area of residence (near the study site) for the full duration of the study, so it is convenient for them to attend study visits at the site.

The following conditions ARE NOT CONSIDERED EXCLUSION CRITERIA. Therefore, candidates complying with them ARE ELIGIBLE FOR INCLUSION in the study:

- Coinfection with others HPV genotypes
- History of cervical surgery.
- History of condyloma acuminata
- History of HIV or other immunosuppressive conditions either acquired or medically induced.
- History of transplant immunosuppression.
- History of an autoimmune disease.

5.2 EXCLUSION CRITERIA

5.2.1 Medical Conditions

(The history of medical conditions will be based on the self-report or medical chart provided by the subject)

Candidates must be excluded from the study if they:

- Have any cervical lesion that requires clinical intervention within 7 months that could significantly affect cervical epithelia (and therefore, HPV viral production), such as cervical conisation (RIFT-HPV Cohort 1).
- Have a fever (defined as temperature ≥37.8°C) within the 24-hour period prior to the Day 1 visit (Visit 1)*.
- Have a history of severe allergic reaction (e.g., swelling of the mouth and throat, difficulty breathing, hypotension, or shock) that required medical intervention.
- Are allergic to any vaccine component, including aluminium, yeast, or BENZONASETM (nuclease, Nicomedia [used to remove residual nucleic acids from this and other vaccines]). For this exclusion criterion, an allergy to vaccine components is defined as an allergic reaction that met the criteria for severe adverse event (SAE), defined as any untoward medical occurrence that results in death, is life-threatening, requires hospitalization or results in persistent or significant disability/incapacity.
- Have known thrombocytopenia or any coagulation disorder that would contraindicate intramuscular injection of study vaccine.
- Have a history of splenectomy.
- Have a history of ano-genital cancer or HPV-related head and neck cancer.
- Are pregnant at the time of signing informed consent, are planning to become pregnant within the full duration of the study.
- Have a history or current evidence of any condition, therapy, lab abnormality or other circumstance that might confound the results of the study or interfere with the subject's compliance of study procedures for the full duration of the study, such that their inclusion in the study is not in the best interest of the subject and/or may compromise fulfilment of study's objectives, by judgement of the Investigator.
- Are, at the time of signing informed consent, using recreational or illicit drugs or have had a recent history (within the last year) of drug or alcohol abuse or dependence at the discretion of the Investigator that might interfere with her capacity to comply with study procedures. Alcohol abusers are defined as those who drink despite recurrent social, interpersonal, and/or legal problems because of alcohol use.

*: For items denoted with an asterisk it has to be comply before all visits if the exclusion criterion is met, then the visit may be rescheduled for a time when the criterion is not met.

5.2.2 Prior/Concomitant Therapy

Candidates must be excluded from the study if they:

- Have previously received any HPV vaccine.
- Have received within the 3 months prior to the Day 1 vaccination, are receiving, or plan to receive during Day 1 through Month 7 of the study, any immune globulin product (including RhoGAM™ [Ortho-Clinical Diagnostics]) or blood-derived product other than IVIG.
- Have received inactivated or recombinant vaccines within 14 days prior to Day 1 vaccination, or live vaccines within 21 days prior to Day 1 vaccination*.

*: For items denoted with an asterisk it has to be comply before all visits if the exclusion criterion is met, then the visit may be rescheduled for a time when the criterion is not met.

5.2.3 Prior/Concurrent Clinical Study Experience

Candidates must be excluded from the study if they:

- Are concurrently enrolled in other clinical studies of investigational agents.

5.2.4 Sexual Activity

Candidates must be excluded from the study if they:

- Have engaged in sexual activity 48 hours prior to Day 1 (this may result in the detection of viral DNA that has been deposited in the oral cavity and is not the result of ongoing infection) * Sexual activity is defined as:
  - Penile penetrative vaginal intercourse.
  - Penile penetrative anal intercourse.
  - Oral sex involving any contact between subject's mouth with a partner's genital or anal area.

  *: For items denoted with an asterisk it has to be comply before all visits, if the exclusion criterion is met, then the visit may be rescheduled for a time when the criterion is not met.

5.2.5 Other Exclusions

Candidates must be excluded from the study if they:

- Are unlikely to adhere to the study procedures, keep appointments, or are planning to permanently relocate from the area prior to the completion of the study or to leave for an extended period when study visits would need to be scheduled.

5.3 SUBJECT REPLACEMENT STRATEGY

A subject who discontinues from study vaccination or withdraws from the study will not be replaced.

6 STUDY INTERVENTION

6.1 STUDY VACCINE CHARACTERISTICS

Vaccine Name: Nonavalent HPV vaccine (9vHPV/Gardasil-9™).

Dose Formulation: Sterile Suspension.

Unit, Dose, Strength: 0.5 ml.

Dosage Level: HPV 6/11/16/18/31/33/45/52/58 L1 VLP, 30/40/60/40/20/20/20/20/20 mcg per dose.

Route of Administration: Intramuscular.

Vaccination Regimen Use: Day 1, Month 2, Month 6.

Sourcing: Provided centrally by manufacturer through a designated distributor.

Indication: indicated in individuals from 9 years of age for the prevention of diseases caused by vaccine's 9 HPV types: genital warts (HPV6 and 11) and premalignant lesions and cancers affecting the cervix, vulva, vagina and anus (HPV 16, 18, 31, 22, 45, 52 and 58)[9].

Alignment with 9vHPV Summary of Product Characteristics: Administration of 9vHPV to adult women as specified in the inclusion criteria (section 5.1 of this protocol) will be done in compliance with the study vaccine's Summary of Product Characteristics provided by the European Medicines Agency (EMA)[9].

Benefits from study participation: Since eligible subjects are not included in the National HPV Immunization Programme, they will benefit from participating in the study by receiving 9VHPV free of charge.

6.2 SAFETY INFORMATION REFERENCE

All safety information regarding administration of 9vHPV can be found in the Summary of Product Characteristics provided by the European Medicines Agency (EMA)[9].

6.3 PREPARATION, HANDLING, STORAGE, AND ACCOUNTABILITY

6.3.1 Dose Preparation

The designated study personnel (study nurse/sub-investigator) will prepare vaccine doses according to instructions in 9vHPV Summary of Product Characteristics[9].

6.3.2 Handling, Storage, and Accountability

The study's designated Pharmacist in the Pharmacy Unit of the study site will confirm that appropriate temperature conditions have been maintained during transit for all study vaccine doses received, and that any discrepancies are reported and resolved before dose administration.

Only study subjects will receive study vaccine, and only authorized study personnel may supply or administer study vaccine. All study vaccine doses must be stored in a secure, environmentally controlled, and monitored (manual or automated) area in accordance with the labelled storage conditions with access limited to the Investigator and authorized personnel.

The study Pharmacist is responsible for study vaccine accountability, reconciliation, record maintenance, and documented discard and destruction if appropriate.

The study Pharmacist and Principal Investigator are responsible for, and shall take all steps to maintain, appropriate records and ensure appropriate supply, storage, handling, distribution, and usage of study vaccine in accordance with the protocol and any applicable laws and regulations.

6.4 CONCOMITANT THERAPY

Medications or vaccinations specifically prohibited in the exclusion criteria are not allowed during time periods specified by this protocol for that medication or vaccination.

If there is a clinical indication for any medications or vaccinations specifically prohibited, discontinuation from study treatment may be required.

The final decision on any supportive therapy or vaccination rests with the Investigator and/or the subject's primary physician. However, the decision to continue the subject on study treatment requires the mutual agreement of the Investigator, the Sponsor, and the subject.

6.4.1 Specific Restrictions for Concomitant Therapy or Vaccination

If possible, subjects should not receive:

- immune globulins and blood products from Day 1 through Month 7,
- non-study inactivated or recombinant vaccines from 14 days prior to each study vaccination through 14 days after each study vaccination,
- or non-study live vaccines from 21 days prior to each study vaccination through 14 days after each study vaccination.
- Subjects may receive allergen desensitization therapy and tuberculin skin testing while participating in the study.

6.4.2 Rescue Medications and Supportive Care

No rescue or supportive medications are specified for use in this study.

6.4.3 Dose Modification

No dose or regimen modification is allowed in this study.

7 DISCONTINUATION OF STUDY TREATMENT AND SUBJECT WITHDRAWAL

7.1 DISCONTINUATION OF STUDY TREATMENT

As certain data beyond study treatment discontinuation may be important to the study, they should be collected even if the subject has decided to discontinue.

Therefore, subjects discontinuing 9vHPV vaccination prior to completion of the protocol-specified vaccination regimen will be asked to continue in the study as specified in Section 1.2 and Section 8.5.

Subjects may discontinue vaccination at any time for any reason or be discontinued at the discretion of the Investigator should any untoward effect occur. In addition, a subject may be discontinued by the Investigator or the Sponsor if study vaccination is inappropriate, the study plan is violated, or for administrative and/or other safety reasons.

A subject must be discontinued but continue to be monitored in the study for any of the following reasons:

- The subject or subject's legally acceptable representative requests to discontinue.
- The subject has a medical condition or personal circumstance which, in the opinion of the Investigator and/or Sponsor, placed the subject at unnecessary risk from continued administration of study vaccination.

If the subject becomes pregnant throughout the duration of the study, vaccination will be discontinued and resumed after childbirth. The subject may continue with other study procedures at the discretion of the Investigator.

Discontinued subjects may resume study vaccination regime if considered clinically safe and appropriate by the Investigator.

7.2 WITHDRAWAL FROM THE STUDY

A subject will be withdrawn from the study if the subject or subject's legally acceptable representative withdraws consent from the study.

If a subject withdraws from the study, they will no longer receive study vaccination or be followed at scheduled protocol visits.

7.3 LOST TO FOLLOW-UP

If a subject fails to return to the site for a required study visit and/or if the Investigator is unable to contact the subject, the following procedures will be performed:

- The Investigator or designee must attempt to contact the subject and reschedule the missed visit. If the subject is contacted, the subject should be counselled on the importance of maintaining the protocol-specified visit schedule.
- The Investigator or designee must make every effort to regain contact with the subject at each missed visit (e.g., telephone calls and/or a certified letter to the subject's last known mailing address or locally equivalent methods). These contact attempts should be documented in the subject's medical record.

8 STUDY ASSESSMENTS AND PROCEDURES

Study procedures and their timing are summarized in the schedule of activities (SoA, section 1.2.2)

Adherence to the study design requirements, including those specified in the SoA, is essential and required for study conduct.

The Investigator is responsible for ensuring that procedures are conducted by appropriately qualified (by education, training, and experience) staff.

All study-related medical decisions will be made by an Investigator/Subinvestigator who is a qualified physician.

All screening evaluations will be completed and reviewed to confirm that potential study subjects meet all eligibility criteria.

Procedures conducted as part of the subject's routine clinical management and obtained before signing of ICF may be utilized for screening or baseline purposes provided the procedure met the protocol-specified criteria and were performed within the time frame defined in the SoA (section 1.2.2).

Repeat or unscheduled samples may be taken for safety reasons or for technical issues with the samples.

8.1 ADMINISTRATIVE AND GENERAL PROCEDURES

8.1.1 Informed Consent

Consent must be documented by the subject's dated signature or by her legally acceptable representative's dated signature on an Informed Consent Form (ICF) along with the dated signature of the person conducting the consent discussion (study Investigator or designee).

A copy of the signed and dated ICF should be given to the subject before being included in the study.

The initial ICF, any subsequent revised written ICF, and any written information provided to the subject must receive the Independent Ethics Committee's (IEC's) approval/ favorable opinion in advance of use. The subject or her legally acceptable representative should be informed in a timely manner if new information becomes available that may be relevant to her willingness to continue participation in the study. The communication of this information will be provided and documented via a revised ICF or addendum to the original ICF that captures the subject's or her legally acceptable representative's dated signature.

8.1.2 Inclusion/ Exclusion Criteria Review

All inclusion and exclusion criteria will be reviewed by the study Investigator/Subinvestigator, who is a qualified physician, to ensure that the subject qualifies for being included in the study.

8.1.3 Questionnaire

Study subjects will be required to answer a short questionnaire to obtain relevant data for study's statistical analyses. The questionnaire contains 10 questions requesting information about previous and current health status, HPV infections and vaccination, contraception use and sexual activity.

A study's questionnaire basic form is included in the Annex section 10.1.

8.1.4 Medical History

On Visit 1, the subject's lifetime medical history including history of HPV-related diagnoses will be obtained by the Investigator/Subinvestigator.

After Visit 1, any new medical history that has not been previously documented (i.e., incident medical conditions not considered injection-site AEs, systemic AEs or SAEs) will be collected in the study subject's medical record.

8.1.5 Prior and Concomitant Medications Review

The Investigator or qualified designee will review subject's prior medications or vaccinations on Visit 1. A subject receiving any of the prior medications or vaccinations prohibited in the exclusion criteria (Section 5.2) should not be enrolled into the study. Prior and concomitant medicines or vaccinations should be documented in the data collection system per the following timeframe:

- Immune globulins and blood-derived products, from 3 days prior to Visit 1 through Visit 4.
- Other medications (not specified in the exclusion criteria) from 3 days prior to each study vaccination through 14 days after each study vaccination.
- Non-study inactivated or recombinant vaccines, from 14 days prior to each study vaccination through 14 days after each study vaccination.
- Non-study live vaccines, from 21 days prior to each study vaccination through 14 days after each study vaccination.

The Investigator/Subinvestigator will record concomitant medications or vaccinations, if any, taken by the study subject during study visits' time frames specified above.

For a specific case where a subject mistakenly receives any non-study HPV vaccines only, the non-study HPV vaccine must be reported in the study data collector, regardless of when the non-study HPV vaccine was received.

Study subjects may receive allergen desensitization therapy and tuberculin skin testing while participating in the study.

8.1.6 Study Data Anonymization

All subjects signing the Informed Consent Form will be given a unique Study Identification Number (SIN), for purposes of identification in all procedures thorough the study, and anonymization for data analysis.

Each study subject will be assigned a unique number, and in the event of subject's withdrawal from the study, numbers will not be re-used or reassigned to another participant.

A list of study subjects' IDs and corresponding SINs will be kept by the study site under strict confidentiality in a secured digital environment.

8.1.7 Study Data Management

Study database will be kept at all times in the study site's designated digital store/internal server, which will be secured from unauthorized entry. Access will be exclusively permitted to study Sponsor and Statistician using password encryption.

8.1.8 Study Vaccine Administration

Study vaccine will be prepared and administered by appropriately qualified study personnel (study nurse) as allowed by local/state, country, and institutional guidance.

Adequate vaccine provision, including epinephrine and equipment for maintaining an airway should be available for immediate use should an anaphylactic or anaphylactoid reaction occur.

8.1.8.1 Preparation and Administration of Study Vaccine

Study vaccine must be stored at 2.0°C to 8.0°C protected from light, and it MUST NEVER FREEZE.

Study vaccine must be used as supplied (no dilution before administration). The vaccine vial should be thoroughly mixed before administration by gently rolling the vial between the palms of both hands for 30 seconds before withdrawing the suspension with a syringe.

Study vaccine should be a whitish, semi-translucent suspension when thoroughly mixed. If its appearance is otherwise, the dose will not be administered, and the manufacturer/provider will be contacted immediately.

Study vaccine will be administered in a 3-dose regimen at Day 1/ Visit 1, Month 2/ Visit 2 (±3 weeks), and Month 6/ Visit 3 (±4 weeks).

The 0.5ml dose contained in a single-dose vial should be withdrawn entirely using a sterile needle and syringe and administer promptly by intramuscular injection. The deltoid muscle of the non-dominant arm is the preferred site of vaccination. Study vaccine should not be administered in the buttocks area or within 2 cm of a tattoo, scar, or skin deformation.

Injections should be administered at a 90° angle into the muscle tissue using a needle long enough to ensure intramuscular deposition of study vaccine. The study vaccine should be administered in the deltoid muscle using preferably a 1.0 mL syringe (the largest allowable size is a 3.0 mL syringe) with the following needle length and gauge specifications:

- 1-inch needle, 22 to 23 gauge for subjects weighing <200 pounds (<90.9 kg).
- 1½-inch needle, 22 to 23 gauge for subjects weighing ≥200 pounds (≥90.9 kg).
- 1½ -inch needle, 22 to 23 gauge for thigh injections.

8.1.8.2 Observation Period After Vaccination

All subjects will be observed by the Investigator or qualified designee for at least 30 minutes after each study vaccination for any untoward effects, including allergic reactions. This observation period will be documented in the participant's study chart.

8.1.9 Discontinuation and Withdrawal from the Study

Study subjects who discontinue study intervention prior to completion of the vaccination regimen should be encouraged to continue to be followed for the remaining study visits as outlined in the SoA (section 1.2.2).

When a subject withdraws from participation in the study, all applicable activities scheduled for the final study visit (Visit 4) should be performed at the time of withdrawal. Any AEs that are present at the time of withdrawal should be followed in accordance with the safety requirements outlined in Section 8.4.

8.1.10 Calibration of Equipment

The Investigator or qualified designee has the responsibility to ensure that any device or instrument used for any clinical/laboratory assessment or study medication storage during the study must be adequately calibrated and/or maintained to ensure that the data obtained are reliable and reproducible. Documentation of equipment calibration must be retained as source documentation at the study site.

The following equipment will be used in study procedures and therefore appropriately calibrated and maintained:

- 4ºC refrigerator at site's Pharmacy for vaccine storage.
- -80ºC freezer at site for sample storage.

8.2 STUDY ASSESSMENTS

8.2.1 Sample Collection

8.2.1.1 Cervical and Anal Sample Collection

One cervical and one anal sample will be collected from study subjects on each of the four study visits for assessment of infectivity in HaCaT essay, HPV DNA testing and genotyping and detection of anti-HPV antibodies. On Visits 1 to 3, cervical and anal samples will be collected before administration of the study vaccine's dose.

Cervical samples will be collected using an endocervical brush and the same procedure established for HPV-based screening tests. Anal samples will be collected using a PBS wetting cytobrush. It will be inserted into the anal canal, dep enough to ensure scrapping of both rectal columnar and anal squamous cells.

Once collected, cervical and anal samples will be immediately immersed in a sterile collection tube containing 5 ml of PBS and stored at 4-10 ºC for short term preservation until start of lab procedures (within 48h of collection), or at -80ºC for long-term storage.

8.2.1.2 Oral Rinse and Gargle (ORG) Sample Collection

One ORG sample will be collected from study subjects on each of the four study visits for assessment of infectivity in HaCaT essay, HPV DNA testing and genotyping and detection of anti-HPV antibodies. On Visits 1 to 3, ORG sample will be collected before administration of the study vaccine's dose.

Samples will be collected by a 15-second rinse and 15-second gargle with 10 ml of phosphate-buffered saline solution (PBS) which will preserve physiological conditions of the specimen and thus will not alter antibody-virion interaction in posterior study lab assessments.

Once collected, ORG samples will be immediately placed in ice and stored at 4-10 ºC for short term preservation until start of lab procedures (within 48h of collection), or at -80ºC for long-term storage.

8.2.1.3 First-Void Urine Sample Collection

One first-void urine sample will be collected from study subjects on each of the four visits for assessment of infectivity in HaCaT assay, HPV DNA testing and genotyping and detection of anti-HPV antibodies. On Visits 1 to 3, first-void urine sample will be collected before study vaccine's dose administration.

First-void urine sample will be collected using a Coli-Pee collector after at least 2h without voiding.

Once collected, urine samples will be stored at 4-10 ºC for short-term preservation until start of lab procedures (within 48h of collection), or at -80ºC for long-term storage.

8.2.1.4 Vulvar Sample Collection

One vulvar sample will be collected from study subjects on each of four visits for assessment of infectivity in HaCaT assay, HPV DNA testing and genotyping and detection of anti-HPV

antibodies. On Visits 1 to 3, vulvar sample will be collected before study vaccine's dose administration.

Vulvar sample will be collected using a sterile dry swab DELTALAB supplied in shockproof round bottom polypropylene tube, rub the swab tip across the vulva several times [56].

Once collected, vulvar samples will be stored at 4-10 ºC for short-term preservation until start of lab procedures (within 48h of collection), or at -80ºC for long-term storage.

8.2.1.5 Blood Sample Collection

A blood sample for serum anti-HPV antibody testing will be collected from study subjects on each of the four study visits. On Visits 1 to 3, blood sample will be collected before administration of the study vaccine's dose.

Blood will be extracted in a red-top vacutainer tube by standard venipuncture procedures, mixed by gently inverting the tube 5 times, left at room temperature for coagulation between 30 and 60 minutes, and centrifuge at 1500 g for 15 minutes to separate serum from coagulated blood. After centrifugation, serum (top layer) will be carefully collected with a pipette into a new tube and immediately placed in ice until stored at -80ºC.

Serum samples will be shipped in dry ice to the designated laboratory for anti-HPV L1 antibody titration (cLIA).

8.2.2 Laboratory Procedures

8.2.2.1 HPV Infective Capacity In-Vitro Evaluation: E1^E4-HaCaT Essay

The functional HaCaT-E1^E4 model will be used to assess and quantify the infectivity of body fluids samples from HPV-positive study subjects[29,30]. Briefly, the assay consists in quantifying the levels of HPV E1^E4 mRNA present in HaCaT cell cultures exposed to HPV-positive exudates, since the expression of HPV E1^E4 mRNA is a surrogated marker that correlates with the number of keratinocytes that have been infected.

For the analyses, 20,000 HaCaT keratinocytes will be grown in 6-well plates until they reach sub-confluent status. Then, cells will be exposed to 0,5 mL of cervical, anal, urine, vulvar and oral samples or PBS (negative control) for 24 hours. After the incubation period, cultured cells will be washed with PBS buffer and recovered after treatment with trypsin in a 15 mL Falcon tube. Finally, cells will be pelleted by centrifugation and stored at -80ºC until gene expression analyses.

For the assessment of HPV E1^E4 mRNA expression, two different gene expression analyses will be performed: RT-qPCR using TaqMan assays and in situ RNA hybridization using fluorescent RNAscope probes.

Total RNA from samples and negative controls will be isolated using RNeasy kit (Qiagen) and reverse transcribed to cDNA with High-Capacity cDNA Reverse Transcription kit (Applied Biosystems). Quantification of E1^E4 mRNA levels will be done by real-time qPCR on a CFX96 instrument (BioRad) and type-specific TaqMan assays (Applied Biosystems) for HPV 11/16/18/31 genotypes will be used.

TaqMan assays consist of a pair of unlabeled PCR primers and a probe labelled with a fluorophore and a quencher in its ends. As target amplification moves forward, 5'-exonuclease

activity of Taq polymerase degrades the probe annealed to the target sequence, thus releasing the fluorophore. Then fluorescence is proportional to target abundance and can be measured by the CFX96 instrument, obtaining a Ct value that estimates the initial amount of target cDNA copies.

TaqMan assays targeting housekeeping genes B2M and GAPDH will also be used as normalizers for relative quantification by comparison of Ct values.

8.2.2.2 *HPV Test and Genotyping (Anyplex HPV28)*

The Anyplex II HPV28 (Seegene, Inc) will be used for detection and genotyping of HPV DNA in cervical, anal, urine, vulvar and oral samples from the study subjects. The purpose of the test is to distinguish reduction of infectivity due to 9vHPV vaccination from spontaneous clearance of the HPV infection.

Anyplex II HPV28 is a semi-quantitative method based on real-time PCR able to detect and identify 19 high risk-HPV types (16, 18, 26, 31, 33, 35, 39, 45, 51, 52, 53, 56, 58, 59, 66, 68, 69, 73, 82) and 9 lower risk-HPV types (6, 11, 40, 42, 43, 44, 54, 61, 70) with high sensitivity and specificity.

Samples will be subjected to DNA isolation prior to Anyplex II HPV28 assays with automated Maxwell® RSC Tissue DNA Kit (Promega) according to manufacturer instructions. The genotyping assay comprises multiplex real-time PCR reactions with type-specific primers and probes for the detection of all mentioned HPV types. The type-specific probes are each labelled with a different fluorescent label, and the fluorescent emission is captured by the CFX96 thermal cycler (BioRad). Fluorescent signal intensity correlates with the abundance of the target DNA and a Ct value is assigned according to the cycle in which fluorescent signal exceeds a threshold. Positivity for a given HPV type will be considered when Ct<35. Human beta-globin will also be analyzed as internal control of cellularity.

The Anyplex II HPV28 is a validated method and currently in use for clinical diagnosis.

8.2.2.3 *Anti-HPV L1 ab Detection: Competitive Luminex Immunoassay (cLIA) and Immunoglobulin-G Luminex Immunoassay (IgG LIA)*

The 9-valent HPV cLIA will be used as a primary method to evaluate antibodies specific for HPV vaccine-covered types (6, 11, 16, 18, 31, 33, 45, 52, and 58) in serum samples from study's subjects of RIFT-HPV Cohort 1. Antibodies against HPV types 16 and 18 will be also evaluated using ELISA (section 8.2.2.4). The purpose of the assay is to detect these HPV antibodies before and after vaccination with the 9vHPV vaccine.

For the 9-valent HPV cLIA, 9 HPV type-specific, yeast-derived virus-like particles (VLPs) are coupled to 9 distinct Luminex magnetic microspheres, one for each VLP type. Each VLP-coupled microsphere has its own distinct fluorescent dye that can be recognized by excitation with an infrared laser, allowing for the measurement of antibodies against multiple HPV types from a single test of an individual's serum. HPV type-specific monoclonal antibodies labelled with R-Phycoerythrin (R-PE) compete with an individual's serum antibodies for binding to the neutralizing epitopes of the VLPs. The fluorescent signal from the R-PE-labelled, type-specific monoclonal antibodies is inversely proportional to the anti-HPV antibody concentration of a sample[42].

The IgG LIA or total IgG assay is slightly more sensitive than the cLIA and has been previously used as a secondary assay for supportive analyses in several studies with qHPV vaccine to assess antibody persistence[43]. The IgG LIA measures antibodies to the 9 HPV types covered by the 9-valent HPV vaccine (HPV 6, 11, 16, 18, 31, 33, 45, 52, and 58).

For the 9-valent HPV IgG LIA, HPV type-specific VLP-coupled microspheres are incubated with an individual's serum, washed to remove excess sera, and incubated with an R-PE-conjugated goat anti-human IgG antibody. The intensity of the fluorescent signal from the R-PE-labelled conjugate is proportional to the anti-HPV antibody concentration of a sample[42].

For the 9-valent HPV cLIA and IgG LIA, antibody concentrations are derived from a standard curve, which is generated using a reference standard made from a pool of serum from individuals immunized against the nine HPV types. A standard curve for each HPV type is calculated using a weighted 4 parameter logistic curve fit. Results are expressed as milliMerck Units/ml (mMU/mL).

For the 9-valent HPV cLIA and IgG LIA, serostatus cut-offs for each HPV type will be predefined. A study subject will be considered seropositive to a given HPV type at Visit 1 if her anti-HPV titre, as assessed by cLIA/ IgG LIA, is greater than or equal to the corresponding serostatus cut-off for that HPV type. A subject will be considered seronegative to a given HPV type at Visit 1 if her anti-HPV titre, as assessed by cLIA/ IgG LIA, is less than the corresponding serostatus cut-off for that HPV type.

8.2.2.4 HPV16/18 virion detection and antibody titration (ELISA, electronic microscopy)

Enzyme-linked immunosorbent assays (ELISA) will be performed as primary method to detect and quantify HPV16/18 antibodies in serum samples of RIFT-HPV Cohort 1 and RIFT-HPV Cohort 2, and HPV16/18 virions in cervical, anal, urine, vulvar and oral samples from the study subjects. The purpose of the test is to distinguish reduction of infectivity due to 9vHPV vaccination from non-productive HPV infections.

A sandwich ELISA assay will be used to detect the major capsid protein L1 of HPV 16/18 present in serum and in exudates of productive infections. Samples will be centrifuged for 20 minutes at 3000 rpm and supernatants recovered and added to microtiter plates with wells coated with anti-HPV 16/18 L1 capture antibodies. Samples and controls will be incubated for 20 minutes to let the antigens bind to the capture antibodies and afterwards a washing step will be done for removing unbound substances. A reporter antibody conjugated with HRP enzyme will be added for antigen detection and incubated for 1 hour, followed by a washing step to remove the excess of reporter antibody. Finally, a chromogenic HRP substrate will be added, and optical density measured for indirect quantification of bound virions.

Productive infections will be detected through the identification of new viral particles by electron microscopy (EM) in cervical samples and HPV positive oral and anal samples as previously described[44]. EM experiments will be performed using the scientific platform of the University of Barcelona (UB). The EM will offer a direct measure of viral particles to confirm the virus production, while the ELISA test will offer a faster and more sensitive measurement especially in samples in which a low viral load is expected (oral samples).

8.3 STUDY SAFETY ASSESSMENTS

8.3.1 Physical Examination

A physical examination is optional and will be conducted by the Investigator/Subinvestigator as per institutional standard on Visit 1 to determine whether the candidate meets eligibility criteria for enrolment.

Height and weight will be recorded on Visit 1 before administration of study vaccine.

Physical examination details will be recorded in the subject's study chart. Any medical condition identified during physical examination will be documented in the data collection system. The Investigator will pay special attention to clinical signs related to previous serious illnesses.

8.3.2 Pregnancy test

A urine sample will be collected from candidates in Visits 1 to 4, to perform a pregnancy test by the study personnel. A positive result will exclude the candidate from participation in the study.

8.3.3 Temperature Measurements

The Investigator/Subinvestigator or nurse will assess the subject's temperature before study vaccine administered. It is administered on Day 1/ Visit 1, Month 2/ Visit 2, and Month 6/ Visit 3; and if the subject has fever (defined as temperature of ≥37.8°C) within the 24-hour period prior to receiving a study vaccination, she should not receive the study vaccine. The vaccination visit should be rescheduled until after the fever has resolved.

In the 15 days following post vaccination, if a temperature measure indicates a fever (defined as ≥37.8°C), an AE of "fever" must be documented in the subject's study chart.

8.3.4 Post vaccination Observation Period (30 Minutes)

All study subjects will be observed for 30 minutes after each vaccination for any immediate reactions. If any immediate AEs (including allergic reactions) are observed during this period, the event, the time at which the event occurred within this timeframe, any concomitant medications that were administered, and resolution of the event, must be recorded on the subject's study chart as per routine clinical practice.

8.4 ADVERSE EVENTS (AES), ADVERSE REACTIONS (ARs) AND OTHER REPORTABLE SAFETY EVENTS

The study Investigator is responsible for detecting and documenting any event that meets the criteria and definitions of adverse event (AE) or serious adverse event (SAE) per this protocol.

During the conduct of the study, the presence of adverse events, whether serious or not, will be verified according to the definitions below.

8.4.1 Definitions

An **adverse event (AE)** is considered any untoward medical occurrence to the study subject, which does not necessarily have a causal relationship to the study vaccine. An AE can be any unfavorable and unintended sign (including an abnormal laboratory finding), symptom, or disease temporally associated with the study vaccine.

Any medical condition that was present before the study vaccination and that remains unchanged or improves will not be considered or recorded as an AE. A worsening of that medical condition will be considered as an AE.

A **serious adverse event (SAE)** is defined as any untoward medical occurrence that at any dose:

- results in death,
- is life-threatening,
- requires subject hospitalization or prolongation of existing hospitalization,
- results in persistent or significant disability/incapacity,
- or is a congenital anomaly/birth defect.

Life-threatening is defined as the situation in which, in the opinion of physician, the patient would have died if it had not been for a timely therapeutic intervention.

For reporting purposes, any suspected adverse events considered medically important will be classified as serious even if they do not meet the above criteria.

Medically important events are defined as those events that may not be immediately life-threatening or cause death, hospitalization, or disability, but may jeopardize the subject or require medical or surgical intervention to prevent one of the other outcomes listed above.

Examples of such events include allergic bronchospasm requiring intensive treatment at an emergency room or at home, blood dyscrasias or convulsions not requiring hospitalization, or the development of drug dependency or drug abuse.

Medical and scientific judgment should be used to decide if other situations that have resulted in one of the outcomes listed in the above definitions should be reported as a SAE.

Hospitalization or prolongation of an existing hospitalization is a criterion for considering that an AE is serious. Only admission when the patient stays overnight in the hospital should be considered hospitalization. The following situations do not meet the criteria for a SAE:

- If hospitalization or prolongation of hospitalization is required for completing a procedure required by the protocol (for instance day or night visits are performed for biopsies or surgery required by the protocol).
- If hospitalization or prolongation of hospitalization is part of the routine procedure at the site (such as withdrawal of a stent after surgery).
- If hospitalization was scheduled prior to patient entry in the study.
- If hospitalization was scheduled for a pre-existing condition that has not worsened.

An **adverse reaction (AR)** is any noxious and unintended reaction to the study vaccine, regardless of the dose administered.

An **unexpected adverse reaction (UAR**) is defined as any adverse reaction whose nature or severity is not consistent with product information (e.g., Investigator's Brochure for an unapproved investigational drug or the summary of product characteristics for an approved medicinal product)[9].

A **suspected unexpected serious adverse reaction (SUSAR)** is an adverse reaction that is both serious and unexpected.

8.4.2 Attributability Criteria

The causal relationship of the study vaccine to the occurrence of the AE/SAE will be established based on clinical judgment by the study Investigator. For this, other causes will be considered and studied, such as the natural history of underlying diseases, concomitant treatment, other risk factors, and temporal relationship of the event to the study vaccine. In addition, the summary of product characteristics of the study vaccine and concomitant therapy will be reviewed.

To analyze the possible cause-effect relationship, the temporal relationship between vaccine administration and the AE, possible alternative explanations, the outcome (complete remission, partial recovery, death, sequelae, persistence), persistence or not after discontinuation of the study vaccine, recurrence on vaccine rechallenge, or previous knowledge of the event consistent with the known or expected pattern of response to the study drug will be considered.

The causal relationship of an AE to the study vaccine will be described according to the following definitions:

Unlikely related: The adverse event does not occur after a plausible temporal sequence from administration of the study product and/or can be reasonably explained by other factors such as the patient's clinical state, toxic or environmental factors, or other concomitant therapies. In addition, it does not follow the known or expected pattern of response to the drug.

Possible relationship: The adverse event occurs after a plausible temporal sequence from administration of the study product, but can also be explained by the patient's clinical state, toxic or environmental factors, or other concomitant therapies. In addition, it does not follow the known or expected pattern of response to the drug.

Probable relationship: The adverse event occurs after a plausible temporal sequence from administration of the study product, cannot be reasonably explained by the patient's clinical state, toxic or environmental factors, or other concomitant therapies, and after withdrawal or dose reduction of the suspect drug, the event follows a logical clinical sequence. In addition, it follows the known or expected pattern of response to the drug.

Clear relationship: The adverse event occurs after a plausible temporal sequence from administration of study product cannot be reasonably explained by the patient's clinical state, toxic or environmental factors or other concomitant therapies, after withdrawal or dose reduction of the suspect drug, the event follows a logical clinical sequence, and the adverse event recurs after reintroduction of the suspect drug. In addition, it follows the known or expected pattern of response to the drug.

No relationship: The adverse event is clearly due to causes unrelated to the study drug, and the criteria for another causal relationship are not met.

No assessable relationship: Any report suggesting an adverse effect, which cannot be judged because the information is insufficient or contradictory, and which cannot be supplemented or verified.

8.4.3 Detection and Recording of Adverse Events

All AEs will be reported by the study subject to the Investigator at each study visit by open-ended, nonleading verbal questioning, and will be recorded based on subject's reporting, careful clinical observation of the subject and results of laboratory tests.

All AEs (serious or not) occurring during the study will be noted in the subject's medical history and recorded in the study CRF. The Investigator will also note whether the adverse event is, based on his/her judgment, related or not to the study vaccine. This decision should also be noted in the medical history and CRF.

At each visit, all AEs experienced by the patient since the previous visit should be recorded in the specific adverse event form of the CRF.

The following will be recorded for each event:

- description, severity (grade 1, 2, 3, 4 and 5),
- duration (start and end dates),
- causal relationship with the study vaccine (according to the above attributability criteria) for which this causal relationship is suspected,
- treatment (if applicable) or actions taken,
- possible alternative explanations,
- predisposing factors,
- outcome,
- for a pre-existing AE that has worsened in terms of severity or frequency, the meaning of the change should be specified.

AE's severity degree provides a qualitative assessment of the extent or intensity of an adverse event elicited by the Investigator or reported by the subject. Severity does not reflect the clinical seriousness of the event, only the grade or extent of the complaint or incidence.

The severity of an AE will be rated based on the Division of AIDS Adverse Event Grading Table[45].

8.4.4 Expedited Reporting of SAEs by the Investigator

The Investigator will report immediately to the Sponsor/representative all serious adverse events regardless of their degree of causal relationship with the study drug.

All SAEs occurring from signing of informed consent and up to 30 days after receiving the last dose of the study drug should be reported.

The initial report of SAE should be written as complete as possible, including details of the current disease and SAE and assessment of the causal relationship between the SAE and the investigational product.

Reporting will be made using the Serious Adverse Event Report Form within 24 hours from first knowledge by the Investigator, completing all information on the form in the following two days.

The information missing at the time of the initial report must be reported in the SAE follow-up form.

For SAEs, the Investigator will provide the Sponsor/representative with all documentation related to the event (additional laboratory tests, discharge reports, etc.).

The Investigator must also follow up SAEs and similarly report information related to the event until it has subsided, returned to baseline, can be attributed to products other than the study medication or to factors unrelated to conduct of the study, it is unlikely to obtain additional information, or in case of permanent impairment, until the condition stabilizes.

In the event of death, the Investigator must provide the Sponsor/representative, the Ethics Committee (EC) involved, and the relevant regulatory authorities with all additional information requested by them.

8.4.5 Reporting of Safety Events by the Sponsor

It is the responsibility of the Sponsor/representative to communicate any information regarding study subject's safety and/or affecting whatsoever the study's benefit/risk ratio to the Spanish Agency of Medicines and Sanitary Products (AEMPS) and the EC within 15 calendar days of learning the event[46].

The Sponsor will keep a record of all AEs reported by Investigators. These records will be submitted to regulatory authorities when requested[46].

1.1.1.1 Annual Safety Report (DSUR)

The Sponsor/ representative will prepare and timely submit annual safety reports to the regulatory authorities, i.e., the Spanish Agency of Medicines and Sanitary Products (AEMPS) and CE for the whole study's duration. Safety reports content and submission must follow the ICH E2F guidelines[47], and pertinent regulations[46].

1.1.1.2 Safety Report to Study Investigator

It is the responsibility of the Sponsor to communicate any information regarding study subject's safety to the Investigator as soon as possible.

Information regarding study's SUSARs information will be sent to the Investigator annually through a line listing, together with a summary of the data analysis. The Investigator will also be informed throughout the study about all aspects related to safety, including amendments to the protocol due to safety reasons.

8.4.6 Expedited Reporting of SUSARs by the Sponsor

It is the responsibility of the Sponsor/ representative to communicate any SUSAR occurring from the time of subject's inclusion in the study through the complete duration of the study (7 months) to the regulatory authorities, i.e., the Spanish Agency of Medicines and Sanitary Products (AEMPS) through EudraVigilance, and to the competent bodies of the Autonomous Communities in whose territory the study is being conducted[46,48].

According to AEMPS regulations (section 8 of the AEMPS' Instructions for the Conduction of Clinical Trials), it is not necessary for the Sponsor to communicate study SUSARs to the EC[48].

As defined in section 8.4.1, a SUSAR is any adverse event meeting the following three criteria:

- Serious,

- Unexpected
- Associated to the administration of the study vaccine.

The study Sponsor will report the adverse experience to MSD, the study funder, as specified in the study agreement signed with MSD.

The latest deadline for SUSARs reporting will be 15 calendar days from the time the Sponsor becomes aware of the event. For SUSARs causing death or being life-threatening for the subject, the latest deadline will be 7 calendar days. When possible, complete information on the event will be provided in the following 8 days.

Information on adverse events that are not serious or unexpected and on those considered unrelated to the study vaccine will be collected in tabular form at the end of the study.

The Sponsor/representative will keep a record of all AEs reported by Investigators. These records will be submitted to the Spanish Regulatory Agency when requested.

8.4.7 Follow-Up of Adverse Events

It is the responsibility of the Investigator to follow up SAEs, and other reportable safety events, and to report information related to the events until it has subsided, returned to baseline, can be attributed to products other than the study vaccine or to factors unrelated to the conduct of the study, it is unlikely to obtain additional information, or in case of permanent impairment, until the condition stabilizes.

8.4.8 Exposure During Pregnancy and Breastfeeding

Although not considered an adverse event, it is the responsibility of the Investigator to report immediately to the Sponsor/ representative any pregnancy or suspected pregnancy (including positive pregnancy tests regardless of age) occurring throughout the study and within 28 days after the end of the treatment.

According to Gardasil-9™ Summary of Product Characteristics[9], a large amount of data on pregnant women (more than 1000 pregnancy outcomes) indicates no malformative nor foeto/neonatal toxicity of 9vHPV. Animal studies do not indicate reproductive toxicity. However, these data are considered insufficient to recommend use of 9vHPV during pregnancy[9]. Therefore, a specific selection criterion has been defined, excluding candidates who are pregnant or plan to be during the study (section 5.2).

Any pregnancy occurring after subject's inclusion in the study and exposure to any number of vaccine doses will be followed up to delivery for possible adverse events related to malformation or foeto/neonatal toxicity.

The Investigator will follow the female subject until completion of the pregnancy, and must notify UICEC-IDIBELL immediately about the outcome (either normal or abnormal outcome) of the pregnancy (including spontaneous abortion or voluntary termination, details of birth and the presence or absence of any birth defects, congenital abnormalities or maternal and new-born complications) by email or fax, and by sending a completed Pregnancy Notification Form.

If the outcome of the pregnancy was abnormal (e.g., therapeutic abortion), the Investigator should report the abnormal outcome as an AE. If the abnormal outcome meets any of the

serious criteria, it must be reported as an SAE to UICEC within 24 hours of the Investigator's knowledge of the event using the SAE Notification Form.

According to Gardasil-9™ Summary of Product Characteristics, it is safe to use 9vHPV during breastfeeding[9].

8.5 VISIT REQUIREMENTS

8.5.1 Screening Visit: Day 1/ Visit 1.

Inclusion/exclusion criteria in sections 5.1 and 5.2 specifies restrictions and requirements for Day 1. Signing of Informed Consent, screening of candidates and vaccination with the first dose of Gardasil-9™ will be performed on Day 1. See Schedule of Activities in section 1.2.2 for the specific study procedures on Day 1.

8.5.2 Vaccination Visits: Day 1, Month 2 and Month 6.

Prerequisites for the three vaccination visits are the following:

- The subject has not had a fever (defined as temperature ≥37.8°C) within the 24-hour period prior.
- Has not received a non-study inactivated or recombinant vaccine within 14 days prior to any study vaccination visit or a non-study live vaccine within 21 days prior to any study vaccination visit.

If the subject does not meet the requirements listed above, the study visit (including sample collection and study vaccination) should be re-scheduled.

8.5.3 Cervical, Anal, Urine, Vulvar and Oral Sample Collection Visits: Day 1, Month 2, Month 6 and Month 7.

On visits when cervical, anal, urine, vulvar and oral samples will be collected from the study subject, she must not have engaged in sexual activity within the 48 hours prior. See section 5.2.4 for definition of sexual activity.

The purpose of this restriction is to avoid HPV type-specific contamination of collected specimens due to sexual partner's fluids deposition.

If the subject does not meet this requirement, the study visit (including sample collection and study vaccination) should be re-scheduled.

The subject should not have her period to collect the samples. If the subject does not meet this requirement, the study visit should be re-scheduled.

8.5.4 Blood Sample Collection Visits: Day 1, Month 2, Month 6 and Month 7.

As described in the exclusion criteria list (see section 5.2.2), for candidate enrolment in the study she must not have received within the 3 months prior to the Day 1 visit, any immune globulin product or blood-derived product other than IVIG.

This requirement should be fulfilled throughout the whole duration of the study, to avoid possible interactions between immunoglobulin products and study vaccine or study antibody determination.

9 STATISTICAL ANALYSIS PLAN

9.1 Study Objectives, Endpoints and Data Analysis

9.1.1 Primary Objective

9.1.1.1 Objective 1

To demonstrate that vaccination with a 3-dose regimen of 9vHPV will reduce viral infectivity in cervical, anal, urine, vulvar and oral samples from HPV 16/18/16+18-positive women.

9.1.1.1.1 Endpoint

In-vitro infectivity evaluation (by expression of E1^E4 HPV biomarker in HaCaT keratinocytes) of cervical, anal, urine, vulvar and oral samples collected before and after 9vHPV vaccination in a 3-dose regimen.

9.1.1.1.2 Inclusion in the analysis population

The below criteria will be followed to include cases in the analysis population:

Before vaccination (Visit 1):

- HPV16/18/16+18 positive DNA detection and genotyping (using Anyplex HPV28) in collected cervical, anal, urine, vulvar and oral samples.
- HPV16/18 positive virion detection (using ELISA, electronic microscopy) in collected cervical, anal, urine, vulvar and oral samples.

Therefore, cases showing:

- negative HPV16/18 DNA test and/or
- absence of HPV16/18 viral particles,

will not be included in the analysis population.

After vaccination (Visits 2 to 4, Months 2, 6 and 7):

- HPV16/18/16+18 positive DNA detection and genotyping (using Anyplex HPV28) in collected cervical, anal, urine, vulvar and oral samples.
- HPV16/18 positive virion detection (using ELISA, electronic microscopy) in collected cervical, anal, urine, vulvar and oral samples.
- Positive detection of exudated HPV 6/11/16/18/31/33/45/52/58 L1 antibodies in collected cervical, anal, urine, vulvar and oral samples.
- Positive detection/titration of HPV 6/11/16/18/31/33/45/52/58 L1 antibodies in collected serum samples (positive seroconversion).

Therefore, cases showing:

- negative HPV16/18 DNA test and/or
- absence of HPV16/18 viral particles and/or

- absence of exudated HPV L1 antibodies and/or
- absence of positive seroconversion,

will not be included in the analysis population.

9.1.1.2 Data analysis

Differences in the infectivity rate before (Day 1/ Visit 1) and after vaccination (Month 7/ Visit 4) will be compared in cervical, anal, vulvar, urine and oral samples using non-parametric Wilcoxon signed rank test.

For cervical samples, joint and stratified analysis for HPV-RIFT1 and HPV-RIFT2 cohorts will be done.

Oral, vulvar, urine and anal samples included in the HPV-RIFT1 and HPV-RIFT2 cohorts will be analyzed together to obtain the minimal sample size established for this analysis. If sample size would allow stratified analysis for HPV-RIFT1 and HPV-RIFT2 cohorts, it will be performed and submitted.

Sensitivity analysis including only cases showing negative seroconversion before vaccination will be performed.

9.1.2 Secondary Objectives

9.1.2.1 Objective 2

To summarize antibody responses to vaccination (for each of the 9vHPV-covered HPV types (6, 11, 16, 18, 31, 33, 45, 52, and 58)).

9.1.2.1.1 Endpoints

HPV 6/11/16/18/31/33/45/52/58 L1 antibody detection/titration using cLIA/ ELISA in serum samples collected before and after 9vHPV vaccination.

9.1.2.1.2 Inclusion in the analysis population

All cases showing positive seroconversion for HPV16/18/16+18 will be included.

For each HPV type analyzed, proportion of seroconverted cases after 3 vaccine doses (Month 7/ Visit 4) will be provided.

It is expected that 90% of cases show positive seroconversion after 3-dose 9vHPV vaccination.

9.1.2.2 Objective 3

To demonstrate viral infectivity reduction in cervical, oral, urine, vulvar and anal samples from HPV 16/18/16+18-positive women before and after vaccination with 1-dose or 2-dose regimen of 9vHPV.

9.1.2.2.1 Endpoints, inclusion in the analysis population and data analysis

The same endpoint, analysis inclusion criteria and data analysis as in primary objective will be applied for cases showing 1-dose and 2-dose 9vHPV vaccination.

9.1.3 General Considerations on Data Analysis.

Sensitivity analyses using information provided by the study questionnaire about sexual activity, immunological status, concomitant medication and other will be performed when necessary.

Significant difference in the infectivity rate will be accepted with a p-value <0.05.

A partial analysis after completing 50% of the planned recruitment and a global analysis at the end of the 7-month follow-up will be carried out.

9.1.4 Definitions Regarding Data Description and Analysis.

Infectivity rate: The infectivity rate of cervical, anal, urine, vulvar and oral samples collected from study subjects before and after 9vHPV vaccination will be obtained measuring the in-vitro expression of HPV biomarker E1^E4 mRNA in HaCaT keratinocytes incubated during 24h with study samples (quantitative variable). The expression level of a positive control sample and the human gene HPRT1 will be used to normalize the expression measurements. The quantity of HPV-infected keratinocytes (i.e., expressing E1^E4 mRNA) will be directly proportional to the quantity of free HPV viral particles present in samples (i.e. not neutralized by HPV antibodies). Reduction in infectivity will be expressed as the difference between the infectivity rate of cervical, anal, urine, vulvar and oral samples before and after the subject receives 9vHPV vaccination.

HPV persistent infection: an HPV infection of the same genotype and sample type (oral, anal, urine, vulvar or cervical) detected in samples collected at Day1/Visit 1 and Month 7/Visit 4 is defined as persistent infection (qualitative variable: persistent or non-persistent infection).

HPV productive infection: an HPV positive infection that produce new viral particles detected by immunoassays or EM in oral, anal, urine, vulvar or cervical samples is defined as productive infection (qualitative variable: presence or absence of viral particles).

Positive/negative HPV seroconversion: it is defined as the status of study serum samples regarding presence (positive) or absence (negative) of type-specific HPV antibodies. Positive seroconversion may occur after administration of study vaccine, or by subject's natural immunological response to a previous HPV infection. Negative seroconversion may occur when study vaccine fails to trigger an immunological response in the subject. Quantitative results of the HPV antibodies detection test are reported in terms of arbitrary units (milliMerck units/ml). Results may be reported qualitatively as positive or negative seroconversion for type-specific HPV antibodies.

Anti-HPV antibodies exudation: type-specific HPV antibodies will be detected in cervical, anal, urine, vulvar and oral samples (cLIA/ ELISA), and results will be expressed qualitatively as antibody presence or absence.

9.2 Sample Size and Power

9.2.1 RIFT-HPV 1 cohort

The sample size for this cohort has been calculated based on the demonstration of viral infectivity reduction before and after a 3-dose regimen of 9vHPV vaccination in cervical samples as a reference.

Accepting an alpha risk of 0.05 and a beta risk of less than 0.2 in a bilateral contrast, it has been estimated that a minimum of 39 women fulfilling the 3-dose vaccination schedule is needed to detect a difference equal to or greater than a 25% infectivity reduction in cervical samples.

It has been estimated a 40% sampling loss from those study subjects:

- showing an HPV16/18/double 16-18 positive result not confirmed by Anyplex HPV28,
- not fulfilling the 9vHPV 3-dose schedule,
- showing HPV clearance at Month 7 visit,
- showing a non-productive infection before vaccination.

9.2.2 RIFT-HPV 2 cohort

The sample size for this cohort has been calculated based on the demonstration of viral infectivity reduction before and after a 3-dose regimen of 9vHPV vaccination in anal samples as a reference.

Accepting an alpha risk of 0.05 and a beta risk of less than 0.2 in a bilateral contrast, it has been estimated that a minimum of 28 positive women with multiple anogenital lesions, with an HPV-positive anal sample and fulfilling the 3-dose vaccination schedule is needed to detect a difference equal to or greater than a 35% reduction in infectivity in anal samples.

It has been estimated that:

- more than 60% of study subjects with multiple anogenital infection will show HPV positivity in anal samples.
- at least 28% of study subjects with HPV cervical infection will also show anal infection.
- subjects from the RIFT-HPV 1 cohort will also show anal infection, thus increasing the RIFT-HPV 2 cohort size.

Considering these estimations, it is necessary to enroll 30 subjects in RIFT HPV 2 cohort to detect 18 subjects with anal infection, which added to the 10 subjects with anal infection from the RIFT-HPV 1 cohort, gives a total number of 28 subjects needed to evaluate differences in viral infectivity of anal samples.

10 REGULATORY, ETHICAL AND FINANCIAL CONSIDERATIONS

10.1 Code of Conduct

The Investigator will ensure that this study is conducted in accordance with the principles of the Declaration of Helsinki, ICH Guidelines for Good Clinical Practice and local legislation (RD 1090/2015), and in full conformity with relevant regulations[46,49].

The protocol, the Informed Consent Form (ICF), the Participant Information Sheet (PIS) and any applicable documents will be submitted to an appropriate Independent Ethics Committee (IEC) and Regulatory Authority (AEMPS) for written approval. All substantial amendments to the originally approved documents will be also sent to IEC and AEMPS for written approval.

10.2 Financial Support

This study is supported by competitive grants from the Spanish Health Institute Carlos III (code # PI20/01819) and MSD (Investigator-Initiated Study, code # IISP 60552).

10.3 Investigator Financial Disclosure

The Principal Investigator is requested by the Regulatory Authority (AEMPS) and the IEC to provide an accurate disclosure statement of their financial interest that may pose a conflict with the performance of study's procedures and obtention of data according to national ethical regulations and the code of conduct stated in this protocol.

10.4 Payments to site personnel and subjects

There is no economic incentive for any of the personnel included in the study site's Investigator team. Likewise, candidates to be enrolled in the study or enrolled subjects will not receive any economic compensation for their time invested in study's procedures.

10.5 Personal Data Protection

The study site and the Sponsor are responsible for the appropriate processing of the subject's personal data to comply with current legislation on data protection in Spain (Organic Law 3/2018, of December 5th, on Protection of Personal Data and Guarantee of Digital Rights)[50] and in the European Union (Regulation 2016/679 of the European Parliament and of the Council, of April 27, 2016, on the protection of natural persons with regard to the processing of personal data and on the free movement of such data)[51].

The study site and the Sponsor are responsible for the processing of all data generated in the study, according to the obligations derived from their activity in the study:

- The study site is responsible for all data included in medical records, which contains personal data that may identify study subjects.
- The Sponsor is responsible for the codified (anonymized) data.

During the informed consent procedure (Participant Information Sheet and Informed Consent Form), the Investigator will inform candidates to participate in the study of the usage and level of disclosure of their personal study-related data by the Sponsor.

Study candidates will also be informed that their medical records may be examined by authorized personnel appointed by the Sponsor, by Ethics Committee members, and by inspectors from regulatory authorities.

Once enrolled, study subjects will be assigned a unique Study Identification Number (SIN) by the Sponsor, and any records or datasets that are transferred to the Sponsor will contain the subject's SIN only. Any personal information that would make the subject identifiable will not be transferred.

10.6 Study Registration, Results Posting and Publication Policy

The Sponsor will register the study in the Spanish Registry of Clinical Trials (REec), as per local regulations (RD 1090/2015)[46]. The study will also be submitted by the Sponsor to http://www.clinicaltrials.gov.

The study results and reports (positive or negative) will be posted by the Sponsor in the REec, and will be published in scientific journals and/or presented at scientific meetings by the Sponsor in compliance with publication requirements according to standard editorial and ethical practice, and following local regulations (RD 1090/2015) [46].

If publication activity is the initiative of the Investigator, he/she will submit all manuscripts or abstracts to the Sponsor before submission. This allows the Sponsor to protect proprietary information and to provide comments.

Authorship will be determined by mutual agreement and in line with International Committee of Medical Journal Editors authorship requirements.

10.7 Management of Biological Samples

Collection of biological samples for evaluation of study endpoints will be performed according to protocol specifications (section 8.2.1). Samples will be collected from enrolled subjects during study visits by the Investigation team (Investigator/ Nurse) and processed and/or stored by the Sponsor according to per-protocol laboratory assessments (section 8.2.2).

After completing all analyses, samples surplus will be stored by the Sponsor in adequate conditions to preserve their integrity, so they can be used in future investigations. Express consent from study subjects for future use of samples will be requested, by checking a YES or NO box in the study's informed consent during Visit 1.

Samples will be registered in collection C.0006798 of the National Registry of Biobanks, for which the study Sponsor is responsible. Samples will not be transferred to third parties, and will be used in future studies on the diagnosis and prevention of HPV infections and related diseases, which have been approved by a Research Ethics Committee. Use of samples for purposes unrelated to those specified in this section will require express reconsent from study subjects.

11 ANNEX

11.1 STUDY QUESTIONNAIRE

RIFT-HPV QUESTIONNAIRE

Visit date: ______/______/______

Visit 1/ Day 1 Visit 2/ Month 2 Visit 3/ Month 6 Visit 4/ Month 7

PERSONAL INFORMATION

Name:

____

Birth date: ______/______/______

Gender □ Male □ Female □ Other □

Date of last menstruation period:______ /______/_______

Time since last urination (before taking the FV sample):______________________

Time since last thorough douching: _______________________________________
(before taking the FV sample)

HPV-RELATED INFORMATION (before to start the study)

HPV vaccination	None □	2vHPV □	4vHPV □	9vHPV □
HPV disease	None □	Yes: __________________________________		

HEALTH STATUS

Immune disease	None □	Yes:___________________________________
Medication affecting immune system	None □	Yes:___________________________________ _______________________________________ _______________________________________

ORAL CONTRACEPTION

Use of oral contraceptives	None □	Yes: ___________________________________

SEXUAL ACTIVITY (Sexual activity is defined as penile penetrative vaginal intercourse, penile penetrative anal intercourse or oral sex involving any contact between subject's mouth with a partner's genital or anal area)

Did you have practice sex within last 48h? Yes □ No□

Date of the last intersexual course?

SEXUAL ACTIVITY (in the last 6 months/ from last visit)

Total number of sexual partners: ____

Number of sexual partner with whom you have practice:

Penetrative vaginal	Number:___	None □
Penetrative anal	Number: ____	None □
Oral	Number: ____	None □

11.2 SUBJECT'S INFORMATION SHEET AND INFORMED CONSENT FORM (ICF)

Find the Participant's Information Sheet and ICF accompanying this protocol.

11.3 PERSONAL DATA USAGE AUTHORIZATION FORM

Find the study's Personal Data Usage Authorization Form accompanying this protocol.

12 REFERENCES

1 Bosch FX, Broker TR, Forman D, Moscicki A-B, Gillison ML, Doorbar J *et al.* Comprehensive Control of Human Papillomavirus Infections and Related Diseases. *Vaccine* 2013; **31**: G1–G31.

2 Bosch FX, Robles C, Díaz M, Arbyn M, Baussano I, Clavel C *et al.* HPV-FASTER: broadening the scope for prevention of HPV-related cancer. *Nature Reviews Clinical Oncology* 2015; **13**: 119.

3 WHO Director-General calls for all countries to take action to help end the suffering caused by cervical cancer. WHO. 2018.http://www.who.int/reproductivehealth/call-to-action-elimination-cervical-cancer/en/ (accessed 27 May2019).

4 Schiffman M, Doorbar J, Wentzensen N, de Sanjosé S, Fakhry C, Monk BJ *et al.* Carcinogenic human papillomavirus infection. *Nat Rev Dis Primers* 2016; **2**: 16086.

5 Johnson KM, Kines RC, Roberts JN, Lowy DR, Schiller JT, Day PM. Role of heparan sulfate in attachment to and infection of the murine female genital tract by human papillomavirus. *J Virol* 2009; **83**: 2067–2074.

6 Raff AB, Woodham AW, Raff LM, Skeate JG, Yan L, Da Silva DM *et al.* The evolving field of human papillomavirus receptor research: a review of binding and entry. *J Virol* 2013; **87**: 6062–6072.

7 Doorbar J. The E4 protein; structure, function and patterns of expression. *Virology* 2013; **445**: 80–98.

8 de Sanjosé S, Brotons M, Pavón MA. The natural history of human papillomavirus infection. *Best Practice & Research Clinical Obstetrics & Gynaecology* 2018; **47**: 2–13.

9 European Medicines Agency. Gardasil 9-Product Information. 2020.https://www.ema.europa.eu/en/medicines/human/EPAR/gardasil-9 (accessed 16 Feb2021).

10 Roden RBS, Stern PL. Opportunities and challenges for human papillomavirus vaccination in cancer. *Nat Rev Cancer* 2018; **18**: 240–254.

11 Klasse PJ. Neutralization of Virus Infectivity by Antibodies: Old Problems in New Perspectives. *Adv Biol* 2014; **2014**. doi:10.1155/2014/157895.

12 Mallery DL, McEwan WA, Bidgood SR, Towers GJ, Johnson CM, James LC. Antibodies mediate intracellular immunity through tripartite motif-containing 21 (TRIM21). *Proc Natl Acad Sci U S A* 2010; **107**: 19985–19990.

13 Castellsagué X, Muñoz N, Pitisuttithum P, Ferris D, Monsonego J, Ault K *et al.* End-of-study safety, immunogenicity, and efficacy of quadrivalent HPV (types 6, 11, 16, 18) recombinant vaccine in adult women 24–45 years of age. *British Journal of Cancer* 2011; **105**: 28–37.

14 Kemp TJ, García-Piñeres A, Falk RT, Poncelet S, Dessy F, Giannini SL *et al.* Evaluation of systemic and mucosal anti-HPV16 and anti-HPV18 antibody responses from vaccinated women. *Vaccine* 2008; **26**: 3608–3616.

15 Castle PE, Rodriguez A-C, Bowman FP, Herrero R, Schiffman M, Bratti MC *et al.* Comparison of ophthalmic sponges for measurements of immune markers from cervical secretions. *Clin Diagn Lab Immunol* 2004; **11**: 399–405.

16 Schwarz TF, Kocken M, Petäjä T, Einstein MH, Spaczynski M, Louwers JA *et al.* Correlation between levels of human papillomavirus (HPV)-16 and 18 antibodies in serum and cervicovaginal secretions in girls and women vaccinated with the HPV-16/18 AS04-adjuvanted vaccine. *Hum Vaccin* 2010; **6**: 1054–1061.

17 Parker KH, Kemp TJ, Pan Y, Yang Z, Giuliano AR, Pinto LA. Evaluation of HPV-16 and HPV-18 specific antibody measurements in saliva collected in oral rinses and merocel® sponges. *Vaccine* 2018; **36**: 2705–2711.

18 Hagensee ME, Koutsky LA, Lee SK, Grubert T, Kuypers J, Kiviat NB *et al.* Detection of cervical antibodies to human papillomavirus type 16 (HPV-16) capsid antigens in relation to detection of HPV-16 DNA and cervical lesions. *J Infect Dis* 2000; **181**: 1234–1239.

19 Dillner L, Bekassy Z, Jonsson N, Moreno-Lopez J, Blomberg J. Detection of IgA antibodies against human papillomavirus in cervical secretions from patients with cervical intraepithelial neoplasia. *Int J Cancer* 1989; **43**: 36–40.

20 Wang Z, Hansson BG, Forslund O, Dillner L, Sapp M, Schiller JT *et al.* Cervical mucus antibodies against human papillomavirus type 16, 18, and 33 capsids in relation to presence of viral DNA. *J Clin Microbiol* 1996; **34**: 3056–3062.

21 Loimaranta V, Sievi K, Werner J, Pawlita M, Waterboer T, Butt J *et al.* Comparison of multiplex-serology and ELISA based methods in detecting HPV16 L1 antibody responses in paired saliva and serum samples of healthy men. *J Virol Methods* 2019; **270**: 26–33.

22 Cameron JE, Snowhite IV, Chaturvedi AK, Hagensee ME. Human papillomavirus-specific antibody status in oral fluids modestly reflects serum status in human immunodeficiency virus-positive individuals. *Clin Diagn Lab Immunol* 2003; **10**: 431–438.

23 Broich G, Sasaki T. Electron microscopic detection of human papillomavirus particles in oral proliferative lesions. *Bull Tokyo Dent Coll* 1989; **30**: 213–220.

24 Hills E, Laverty CR. Electron microscopic detection of papilloma virus particles in selected koilocytotic cells in a routine cervical smear. *Acta Cytol* 1979; **23**: 53–56.

25 Wissing MD, Burchell AN, El-Zein M, Tellier P-P, Coutlée F, Franco EL. Vaccination of Young Women Decreases Human Papillomavirus Transmission in Heterosexual Couples: Findings from the HITCH Cohort Study. *Cancer Epidemiol Biomarkers Prev* 2019; **28**: 1825–1834.

26 MacCosham A, El-Zein M, Burchell AN, Tellier P-P, Coutlée F, Franco EL. Transmission reduction and prevention with HPV vaccination (TRAP-HPV) study protocol: a randomised controlled trial of the efficacy of HPV vaccination in preventing transmission of HPV infection in heterosexual couples. *BMJ Open* 2020; **10**. doi:10.1136/bmjopen-2020-039383.

27 Franco DE. Transmission Reduction and Prevention with HPV Vaccination (TRAP-HPV) Study: A Randomized Controlled Trial of the Efficacy of HPV Vaccination in Preventing Transmission of HPV Infection in Heterosexual Couples. clinicaltrials.gov, 2020https://clinicaltrials.gov/ct2/show/study/NCT01824537 (accessed 26 Apr2021).

28 Doorbar J. Model systems of human papillomavirus-associated disease. *J Pathol* 2016; **238**: 166–179.

29 Patterson NA, Smith JL, Ozbun MA. Human papillomavirus type 31b infection of human keratinocytes does not require heparan sulfate. *J Virol* 2005; **79**: 6838–6847.

30 Ozbun MA. Infectious human papillomavirus type 31b: purification and infection of an immortalized human keratinocyte cell line. *J Gen Virol* 2002; **83**: 2753–2763.

31 Biryukov J, Myers JC, McLaughlin-Drubin ME, Griffin HM, Milici J, Doorbar J *et al.* Mutations in HPV18 E1^E4 Impact Virus Capsid Assembly, Infectivity Competence, and Maturation. *Viruses* 2017; **9**. doi:10.3390/v9120385.

32 US Food and Drug Administration. Gardasil-9. *FDA* 2020.https://www.fda.gov/vaccines-blood-biologics/vaccines/gardasil-9 (accessed 18 Apr2021).

33 Murillo R, Reyes CO-. Human papillomavirus (HPV) vaccination: from clinical studies to immunization programs. *International Journal of Gynecologic Cancer* 2019; **29**. doi:10.1136/ijgc-2019-000582.

34 Arbyn M, Xu L, Simoens C, Martin-Hirsch PP. Prophylactic vaccination against human papillomaviruses to prevent cervical cancer and its precursors. *Cochrane Database Syst Rev* 2018; **5**: CD009069.

35 Arbyn M, Xu L. Efficacy and safety of prophylactic HPV vaccines. A Cochrane review of randomized trials. *Expert Rev Vaccines* 2018; **17**: 1085–1091.

36 Lei J, Ploner A, Elfström KM, Wang J, Roth A, Fang F *et al.* HPV Vaccination and the Risk of Invasive Cervical Cancer. *New England Journal of Medicine* 2020. doi:10.1056/NEJMoa1917338.

37 Jentschke M, Kampers J, Becker J, Sibbertsen P, Hillemanns P. Prophylactic HPV vaccination after conization: A systematic review and meta-analysis. *Vaccine* 2020; **38**: 6402–6409.

38 Spanish National Immunization Program | vaccination calendar throughout life. 2021.https://www.mscbs.gob.es/profesionales/saludPublica/prevPromocion/vacunaciones/calendario-y-coberturas/docs/CalendarioVacunacion_Todalavida.pdf.

39 Spanish National Immunization Program | vaccination in adults at risk. 2021.https://www.mscbs.gob.es/profesionales/saludPublica/prevPromocion/vacunaciones/calendario-y-coberturas/docs/CalendarioVacunacion_GRadultos.pdf.

40 Human Papillomavirus | immunization guidelines. Ministry of Health, Government of Spain. https://www.mscbs.gob.es/profesionales/saludPublica/prevPromocion/vacunaciones/vacunas/profesionales/vph.htm (accessed 14 Jun2021).

41 Stier EA, Sebring MC, Mendez AE, Ba FS, Trimble DD, Chiao EY. Prevalence of anal human papillomavirus infection and anal HPV-related disorders in women: a systematic review. *Am J Obstet Gynecol* 2015; **213**: 278–309.

42 Opalka D, Matys K, Bojczuk P, Green T, Gesser R, Saah A *et al.* Multiplexed Serologic Assay for Nine Anogenital Human Papillomavirus Types. *Clin Vaccine Immunol* 2010; **17**: 818–827.

43 Kahn J, Xu J, Kapogiannis BG, Sleasman JW. Antibody responses to Quadrivalent HPV Vaccination in HIV-Infected Young Women as Measured by Total IgG and Competitive Luminex Immunoassay. *J Acquir Immune Defic Syndr* 2017; **75**: 241–245.

44 Goldsmith CS, Miller SE. Modern uses of electron microscopy for detection of viruses. *Clin Microbiol Rev* 2009; **22**: 552–563.

45 DAIDS Adverse Event Grading Tables | DAIDS Regulatory Support Center (RSC). https://rsc.niaid.nih.gov/clinical-research-sites/daids-adverse-event-grading-tables (accessed 20 Apr2021).

46 BOE | Real Decreto 1090/2015, de 4 de diciembre, por el que se regulan los ensayos clínicos con medicamentos, los Comités de Ética de la Investigación con medicamentos y el Registro Español de Estudios Clínicos. https://www.boe.es/eli/es/rd/2015/12/04/1090.

47 ICH E2F Development safety update report. European Medicines Agency. 2018.https://www.ema.europa.eu/en/ich-e2f-development-safety-update-report (accessed 30 Sep2021).

48 Documento de instrucciones de la Agencia Española de Medicamentos y Productos Sanitarios para la realización de ensayos clínicos en España. https://www.aemps.gob.es/investigacionClinica/medicamentos/docs/Instrucciones-realizacion-ensayos-clinicos.pdf.

49 ICH E6 (R2) Good clinical practice. European Medicines Agency. 2018.https://www.ema.europa.eu/en/ich-e6-r2-good-clinical-practice (accessed 30 Sep2021).

50 BOE.es - BOE-A-2018-16673 Ley Orgánica 3/2018, de 5 de diciembre, de Protección de Datos Personales y garantía de los derechos digitales. https://www.boe.es/buscar/act.php?id=BOE-A-2018-16673 (accessed 30 Jul2021).

51 Regulation (EU) 2016/679 of the European Parliament and of the Council of 27 April 2016 on the protection of natural persons with regard to the processing of personal data and on the free movement of such data. Publications Office of the European Union. 2016. http://op.europa.eu/en/publication-detail/-/publication/3e485e15-11bd-11e6-ba9a-01aa75ed71a1 (accessed 30 Jul2021).

52 Pattyn J, Panicker G, Willhauck-Fleckenstein M, Van Keer S, Téblick L, Pieters Z, Tjalma W, Matheeussen V, Van Damme P, Waterboer T, Unger E, Vorsters A. Comparison of a VLP-based and GST-L1-based multiplex immunoassay to detect vaccine-induced HPV-specific antibodies in first-void urine. *J Med Virol* 2020; **92**: 3774-3783.

53 Pattyn J, Van Keer S, Téblick L, Van Damme P, Vorsters A. Non-invasive assessment of vaccine-induced HPV antibodies via Firs-void urine. *Front immunol*; **11**:1657.

54 Téblick L, Van Keer S, De Smet A, Van Damme P, Laeremans M, Rios Cortes A, Beyers K, Vankerckhoven V, Mandersloot R, Floore A, Meijer CJLM, Steenbergen RDM, Vorsters A.

Impact of collection volume and DNA extraction method on the detection of biomarkers and HPV DNA in firs-void urine. *Molecules* 2021; **26**: 1989.

55 Khoo SP, Lim WT, Rajasuriar R, Nasir NH, Gravitt P, Woo YL. The acceptability and preferences of vaginal self-sampling for Human Papillomavirus (HPV) testing among a multi-ethnic Asian female population. *Cancer Prev Res* 2021; **14**: 105-12.

56 Sellors JW, Lorincz AT, Mahony JB, Miezynska I, Lytwyn A, Roth P, Howard M, Chong S, Daya D, Chapman W, Chernesky M. Comparison of self-collected vaginal, vulvar and urine samples with physician-collected cervical samples for human papillomavirus testing to detect high-grade squamous intraepithelial lesions. *CMAJ* 2000; **163**: 513-8.

57 Pattyn J, Van Keer S, Tjamal W, Matheeussen V, Van Damme P, Vorsters A. Infection and vaccine-induced HPV-specific antibodies in cervicovaginal secretions. A review of the literature. *Papillomavirus Research* 2019;100185.
