# Supplementary material for: Assessing the reduction of viral infectivity in HPV16/18-positive women after one, two, and three doses of Gardasil-9 (RIFT): Study protocol
Source: PLoS One. 2024 May 20;19(5):e0304080. doi: 10.1371/journal.pone.0304080 (PMC11104652; doi:10.1371/journal.pone.0304080)
Supplement: S3 File — (PDF) [file pone.0304080.s003.pdf]

RIFT-HPV QUESTIONNAIRE

Visit date: ______/______/______

Visit 1/ Day 1 Visit 2/ Month 2 Visit 3/ Month 6 Visit 4/ Month 7

PERSONAL INFORMATION

Name:

____

Birth date: ______/______/______

Gender □ Male □ Female □ Other □

Date of last menstruation period: ______/______/______

Time since last urination: ____
(before urine sample collection)

Time since last thorough douching: _______________________________________
(before urine sample collection)

HPV-RELATED INFORMATION (before RIFT-HPV study)

HPV vaccination	None □	2vHPV □	4vHPV □	9vHPV □
HPV disease	None □	Yes: _________________________________		

HEALTH STATUS

Immune disease	None □	Yes:___________________________________
Medication affecting immune system	None □	Yes:___________________________________<br>______________________________________<br>______________________________________

ORAL CONTRACEPTION

Use of oral contraceptives	None □	Yes: ___________________________________

SEXUAL ACTIVITY (Sexual activity is defined as penile penetrative vaginal intercourse, penile penetrative anal intercourse or oral sex involving any contact between subject's mouth with a partner's genital or anal area)

Did you have practice sex within last 48h? Yes □ No□

Date of last sexual intercourse: ______/______/______

SEXUAL ACTIVITY (in the last 6 months/ from last visit)
Total number of sexual partners: ____

Type of sexual intercourse:

Penetrative vaginal	Yes □	Nº of partners: ____	None □
Penetrative anal	Yes □	Nº of partners: ____	None □
Oral	Yes □	Nº of partners: ____	None □
